# Temporal trends and demographic influences on protein-energy malnutrition in China: a comprehensive analysis from 1990 to 2021

**DOI:** 10.3389/fnut.2025.1583740

**Published:** 2025-05-16

**Authors:** Zhouwei Zhan, Erhan Yu, Rui Huang, Hui Lin, Jiami Yu, Xiaojie Wang, Zengqing Guo, Bijuan Chen

**Affiliations:** ^1^Department of Medical Oncology, Clinical Oncology School of Fujian Medical University, Fujian Cancer Hospital, Fuzhou, Fujian, China; ^2^Department of Neurology, Fujian Medical University Union Hospital, Fuzhou, Fujian, China; ^3^Digestive Endoscopy Center, Clinical Oncology School of Fujian Medical University, Fujian Cancer Hospital, Fuzhou, Fujian, China; ^4^Department of Radiation Oncology, Clinical Oncology School of Fujian Medical University, Fujian Cancer Hospital, Fuzhou, Fujian, China

**Keywords:** protein-energy malnutrition, Global Burden of Disease study, China, disease burden, temporal trends, age-period-cohort analysis

## Abstract

**Background:**

Protein-energy malnutrition (PEM) remains a critical public health challenge, particularly in aging populations and early childhood. Understanding long-term trends in PEM burden is essential for guiding nutritional policy and targeted interventions in China.

**Methods:**

Data were derived from the Global Burden of Disease (GBD) Study 2021 for the period 1990–2021. Age-standardized incidence, prevalence, mortality, disability-adjusted life years (DALYs), years lived with disability (YLDs), years of life lost (YLLs) rate were analyzed using joinpoint regression and age-period-cohort (APC) models to assess temporal trends. Decomposition analysis was employed to attribute changes to population aging, population growth, and epidemiological shifts. Bayesian APC (BAPC) modeling was used to project the PEM burden through 2030 by sex.

**Results:**

In 2021, PEM resulted in over 22 million incident cases and 213,682 DALYs in China, with males exhibiting higher age-standardized rates across all indicators. A bimodal age distribution was observed, with peaks in children under 5 and adults over 80. From 1990 to 2021, incidence and prevalence increased until 2015 and then declined, while mortality and DALYs steadily decreased, particularly among females. Compared to global trends, China demonstrated greater reductions in age-standardized mortality and DALYs, but a slight increase in incidence. Joinpoint analysis revealed critical shifts in trend periods, with marked rises between 2010 and 2015 and subsequent declines. APC modeling indicated increasing burden among recent cohorts aged ≥5 years, despite improvements in young children. Decomposition analysis showed that population growth and epidemiological change were key drivers of incidence and DALYs, while aging reduced both incidence and prevalence in males but increased prevalence in females. BAPC projections suggest continued reductions in all burden indicators through 2030, with a steeper decline in females, although males are expected to maintain a higher burden.

**Conclusions:**

Although the overall health burden of PEM in China has improved, rising incidence and prevalence among older adults highlight the need for age- and sex-targeted nutritional interventions. Proactive strategies are essential to sustain progress and address emerging demographic risks.

## Introduction

Protein-energy malnutrition (PEM) represents a significant public health challenge, particularly in developing countries. PEM, characterized by inadequate intake of protein and calories, manifests in conditions such as marasmus and kwashiorkor and affects both children and adults globally. It is a risk factor for multiple health problems and also predicts poor outcomes in various diseases (1–4). It impairs immune function, increases susceptibility to infections, and can lead to long-term developmental and cognitive impairments in children (5, 6). In China, economic disparities and regional differences significantly influence the prevalence of PEM. Over recent decades, China has undergone substantial socio-economic changes, which have impacted the nutritional status of its population (7). Despite improvements, certain vulnerable groups, including children under five and the older population, remain at high risk (8–10). Children under 5 years are particularly vulnerable, with PEM contributing to stunting, wasting, and underweight conditions, which have long-term implications on cognitive development and economic productivity (11).

The burden of PEM has been extensively studied, revealing that socioeconomic status, healthcare access, and dietary habits are significant determinants of its prevalence. For instance, in regions where maize is a staple, deficiencies in essential amino acids like tryptophan and lysine exacerbate PEM (12). The Global Burden of Disease (GBD) study provides a comprehensive assessment of the burden of PEM by analyzing prevalence, mortality, and disability-adjusted life years (DALYs). The GBD 2021 data offers insights into the epidemiological trends of PEM across different regions and socio-demographic groups, helping to identify high-risk populations and informing public health interventions (13–15). Previous studies have highlighted the persistent burden of PEM in low and middle-income countries, emphasizing the need for targeted nutritional programs and policies (16). In China, despite significant progress in reducing poverty and improving healthcare access, PEM remains a critical issue, particularly in rural areas and among socio-economically disadvantaged groups (17).

Although several studies have described child undernutrition and food insecurity trends (18, 19), few have systematically evaluated PEM across multiple dimensions including incidence, prevalence, and years of healthy life lost in China over the last three decades. This study addresses that gap by analyzing long-term trends in the burden of PEM in China using GBD 2021 data from 1990 to 2021. Through joinpoint regression and age-period-cohort analysis, we identify turning points and generational effects in PEM burden. We further apply decomposition analysis to explore how demographic changes and epidemiological shifts have shaped incidence and outcomes over time. Finally, we use Bayesian age-period-cohort (BAPC) modeling to project incidence, prevalence, and DALYs through 2030. By providing a comprehensive view of PEM dynamics, stratified by age and sex, this study aims to support evidence-based strategies for nutrition surveillance, resource allocation, and targeted intervention in vulnerable populations.

## Methods

### Data sources

This study utilized data from the GBD 2021, a comprehensive, publicly available resource developed by the Institute for Health Metrics and Evaluation (IHME). The GBD framework provides standardized estimates of incidence, prevalence, mortality, and DALYs for a wide range of diseases and injuries across 204 countries and territories, including China, from 1990 to 2021. Estimates are disaggregated by age, sex, location, and year, allowing for detailed temporal and demographic analysis (20, 21). For this study, we extracted data specific to PEM from the GBD Results Tool, including both all-age counts and age-standardized rates (per 100,000 population) for incidence (ASIR), prevalence (ASPR), deaths (ASMR), DALYs, YLDs, and YLLs. All GBD estimates are derived using a rigorous statistical modeling approach that synthesizes data from multiple sources, including household surveys, censuses, disease registries, and vital statistics, adjusted for data quality and comparability across time and geographies. For the China-specific data used in this analysis, primary sources included the China Disease Surveillance Points System, national nutrition and health surveys, population censuses, and other peer-reviewed epidemiological studies. Age-standardization was performed using the GBD world population standard to facilitate cross-population comparisons. All estimates included 95% uncertainty intervals (UIs) generated through 1,000 draws from posterior distributions using Monte Carlo simulations, reflecting uncertainty from both sampling and modeling processes (20, 21).

### Definition and estimation

PEM in the GBD 2021 includes moderate and severe acute malnutrition, commonly referred to as “wasting”, and is primarily defined using weight-for-height Z-scores (WHZ) based on the 2006 World Health Organization (WHO) growth standards for children. PEM is classified into four categories: moderate wasting without edema (WHZ < −2SD to ≥ −3SD), moderate wasting with edema, severe wasting without edema (WHZ < −3SD), and severe wasting with edema. These subtypes enable more granular estimation of health loss related to varying severity of wasting in children. To estimate PEM across all age groups, GBD 2021 uses the DisMod-MR 2.1 Bayesian meta-regression model, which synthesizes data from household surveys, clinical studies, and hospital records. For adults and older children (>5 years), as WHZ scores are not applicable, PEM is identified by International Classification of Diseases (ICD) codes. ICD-10 includes E40 to E46.9 and E64.0, while ICD-9 covers 260–263.9 (14, 20, 21). These codes reflect clinical undernutrition diagnoses. Ensemble modeling ensures internal consistency of estimates across sexes, years, and age groups. To improve pediatric estimation accuracy, GBD revised its ensemble weight-fitting algorithm to prioritize fit within the WHZ distribution that defines child wasting.

### Descriptive analysis

We first performed a descriptive analysis to quantify the burden of PEM in China from 1990 to 2021 using GBD 2021 estimates. Key indicators included the number and age-standardized rates (per 100,000 population) of incidence, prevalence, deaths, DALYs, YLDs, and YLLs. Estimates were stratified by sex, age group, and year to explore population-specific distributions and temporal patterns. Age-standardization was conducted using the GBD global standard population to facilitate comparisons over time and between sexes. In 2021, age- and sex-specific patterns of PEM were visualized to identify population segments with the highest burden, highlighting bimodal distributions in both early childhood and older adults. Temporal trends in all-age numbers and age-standardized rates were also assessed across the 32-year period, with particular attention to turning points in incidence, prevalence, and mortality. Comparisons between China and global data were conducted using extracted GBD metrics to contextualize national trends. All descriptive statistics were accompanied by 95% UIs, reflecting variability across data sources and model simulations, as provided by the GBD methodology (22, 23).

### Joinpoint regression analysis

To identify significant changes in temporal trends of PEM burden in China from 1990 to 2021, we conducted Joinpoint regression analysis using the Joinpoint Regression Program (version 5.2.0) developed by the US National Cancer Institute. This method detects time points (“joinpoints”) at which statistically significant changes in trend occur and calculates the annual percentage change (APC) and average annual percentage change (AAPC) in age-standardized rates across specified segments. Separate models were fitted for each indicator, including ASIR, ASPR, ASMR, age-standardized DALYs, YLDs, and YLLs rate. Analyses were stratified by sex to explore sex-specific trends. The maximum number of joinpoints allowed was set to five, and model selection was based on the Monte Carlo permutation test with a significance level of 0.05 (24). For each segment, the APC and its 95% confidence interval (CI) were reported, with statistical significance defined as a CI that does not include zero.

### Age-period-cohort (APC) analysis

The APC analysis was conducted to disentangle the effects of age, period, and cohort on the incidence, prevalence and DALYs of PEM (25, 26). APC models are useful for understanding temporal trends by accounting for the simultaneous influence of these three factors. B. Carstensen's method from the Lexis diagram was used for APC analysis in this study (27). Data on incidence, prevalence, DALYs and population estimates for each 5-year age group from 1990 to 2021 were obtained from GBD (20). For model fitting, age groups under 5 and over 95 years were grouped together, and age intervals were defined as 0–4, 5–9, 10–14, and so on up to 95–100. For each 5-year period (1992–1996, 1997–2001, etc.), cumulative incidence, prevalence and DALY rates were calculated. APC model fitting was conducted using the Epi package (version 2.51) in R (version 4.3.1). Model fit was evaluated using residual deviations and AIC to determine the optimal model.

### Decomposition analysis

To assess the drivers of change in the burden of PEM in China from 1990 to 2021, we conducted a decomposition analysis to quantify the relative contributions of population growth, aging, and epidemiological changes to the observed differences in incidence, prevalence, and DALYs. This method partitions the total change in burden into demographic and non-demographic components using a counterfactual scenario-based approach (28, 29). Specifically, the total change in PEM burden between 1990 and 2021 was decomposed using a standard three-component demographic decomposition model. First, we calculated the burden that would have occurred in 2021 if only the population size had changed (population growth), keeping the age structure and age-specific rates from 1990 constant. Second, we calculated the effect of changes in population age structure (aging) by applying the 2021 age distribution to the 1990 age-specific rates. The residual difference between the observed 2021 burden and that expected from population growth and aging was attributed to “epidemiological changes”, which reflect shifts in age-specific rates of PEM due to factors such as improved diagnosis, changes in nutritional status, healthcare access, or data reporting practices. This analysis was applied to overall incidence, prevalence, and DALYs, and results were stratified by sex. All decomposition calculations were performed over the full 1990–2021 interval to capture long-term trends.

### Bayesian age-period-cohort (BAPC) analysis

To forecast the future burden of PEM in China, we applied a BAPC model to project trends in age-standardized incidence, prevalence, and DALY rates by sex through 2030. The BAPC model was implemented using the BAPC and INLA packages in R, based on integrated nested Laplace approximations (INLA) for efficient Bayesian inference (30). This approach allows for robust estimation by accounting for temporal dependencies and smoothing effects across age, period, and cohort dimensions, and it is well-suited for public health forecasting using GBD-type longitudinal data. The BAPC model was fitted using GBD 2021 estimates from 1990 to 2021, stratified by sex and 5-year age groups. Projections were generated for 2022 to 2030, and 95% Bayesian credibility intervals (CrIs) were calculated for all forecasted metrics to quantify statistical uncertainty. These CrIs were derived from posterior distributions based on 1,000 simulations and are presented alongside central estimates in figures and tables. In visualizations, shaded bands around projected trend lines represent the 95% CrIs. This modeling approach assumes that historical trends in age, period, and cohort effects will continue into the projection window. While the BAPC framework improves the interpretability and continuity of forecasts, projections remain subject to uncertainty, especially under conditions of sudden policy shifts or emerging nutritional risks. Therefore, interpretation of forecast values should be made with caution, considering the credible intervals as an integral measure of predictive uncertainty.

### Ethics approval

The study did not require ethics approval as it utilized publicly available, de-identified data from the GBD database. The study was conducted in compliance with the Guidelines for Accurate and Transparent Health Estimates Reporting (GATHER) (31).

## Results

### Burden of PEM in China, 2021

In 2021, PEM remained a significant public health concern in China, with notable disparities between sexes. A total of ~22.0 million new PEM cases were reported, with males exhibiting a higher age-standardized incidence rate (1,466.25 per 100,000) than females (1,309.53 per 100,000). The prevalence followed a similar pattern, with over 21.5 million people affected and males again showing a higher age-standardized rate. Although the absolute number of deaths attributable to PEM was relatively low (12,513), the age-standardized mortality rate in males (1.35 per 100,000) was nearly double that in females (0.68 per 100,000). The overall burden, as measured by DALYs, reached 213,682, largely driven by YLLs, which accounted for the majority of DALYs in both sexes. Males bore a disproportionately higher burden, with age-standardized DALY and YLL rates significantly exceeding those in females. Additionally, YLDs contributed minimally to the total burden, but males still experienced a much higher YLD rate (1.21 per 100,000) compared to females (0.10 per 100,000). These findings underscore the persistent burden of PEM in China, particularly among males, and highlight the need for sex-specific nutritional and healthcare strategies (Table 1).

**Table 1:** All-age cases and age-standardized incidence, prevalence, deaths, DALYs, YLDs, and YLLs rates in 2021 for PEM in China.

**Measure**	**All-ages cases**			**Age-standardized rates per 100,000 people**		
	**Total**	**Male**	**Female**	**Total**	**Male**	**Female**
Incidence	22,019,922 (17,990,376, 26,707,208)	11,838,769 (9,768,971, 14,374,588)	10,181,153 (8,279,096, 12,460,987)	1,387.88 (1,133.79, 1,713.44)	1,466.25 (1,202.22, 1,801.02)	1,309.53 (1,060.59, 1,636.17)
Prevalence	21,525,495 (18,105,453, 25,711,670)	11,587,325 (9,738,542, 13,827,501)	9,938,169 (8,399,300, 11,935,991)	1,354.92 (1,151.65, 1,623.8)	1,436.05 (1,217.8, 1,709.32)	1,274.55 (1,072.66, 1,532.78)
Deaths	12,513 (10,400, 14,654)	6,783 (5,604, 8,191)	5,730 (4,356, 7,174)	0.91 (0.75, 1.07)	1.35 (1.13, 1.62)	0.68 (0.52, 0.85)
DALYs	213,682 (180,408, 253,572)	127,443 (104,945, 156,967)	86,240 (68,665, 107,624)	17.54 (14.91, 20.26)	23.56 (19.7, 28.25)	13.29 (10.86, 16.29)
YLDs	9,100 (2,731, 27,957)	8,619 (2,588, 27,316)	480 (82, 1,915)	0.67 (0.26, 1.79)	1.21 (0.43, 3.33)	0.1 (0.03, 0.29)
YLLs	204,582 (173,723, 238,482)	118,823 (98,729, 144,615)	85,759 (68,414, 105,662)	16.87 (14.39, 19.46)	22.36 (18.67, 26.46)	13.2 (10.8, 16.04)

Values in parentheses indicate 95% UIs, estimated using Monte Carlo simulations.

PEM, protein-energy malnutrition; DALYs, disability-adjusted life-years; YLDs, years lived with disability; YLLs, years of life lost.

### Age and sex patterns of PEM in China, 2021

**Figure 1:**
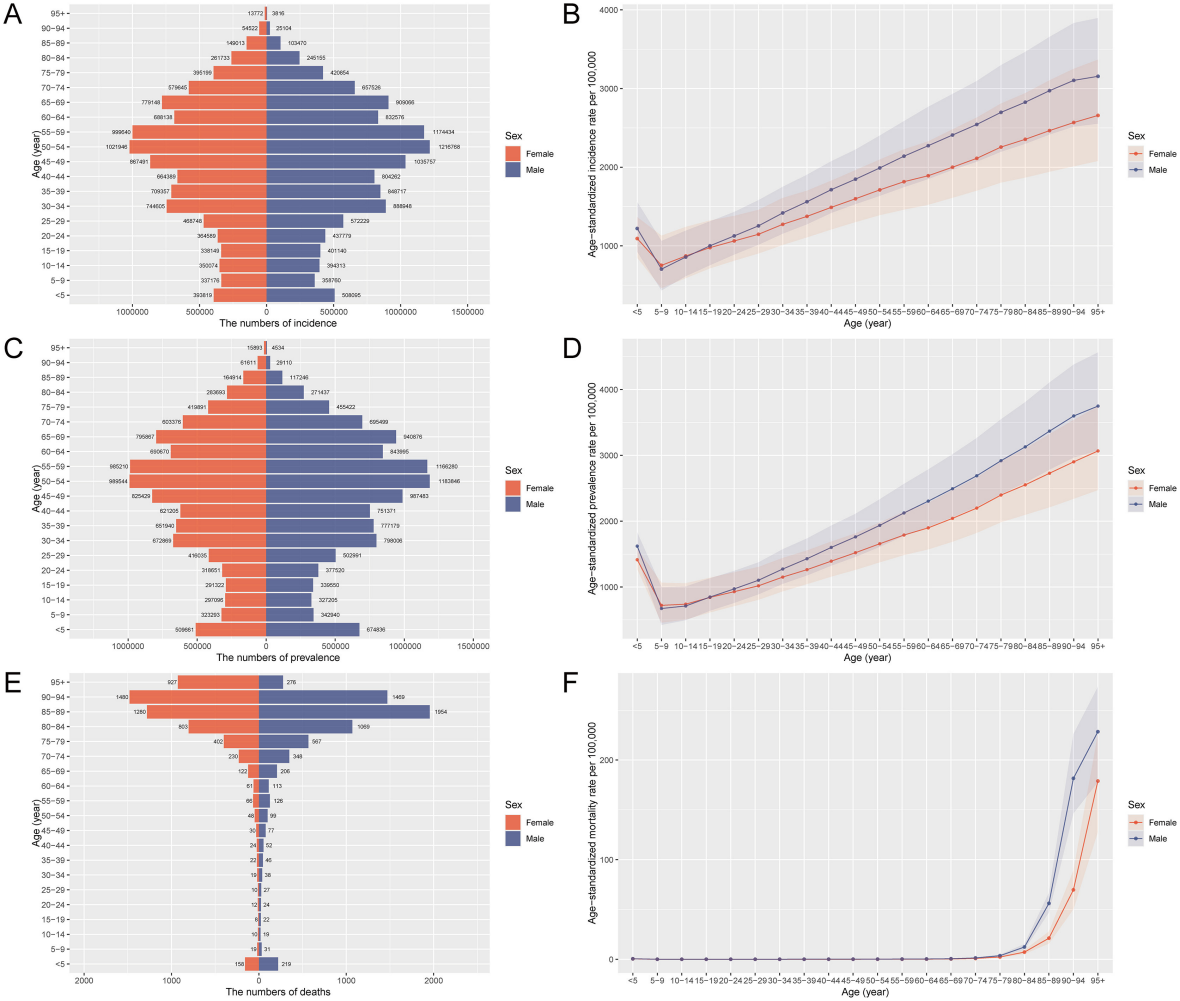
Age and sex distribution of deaths, prevalence, and incidence rates of PEM in China in 2021. **(A)** Age-specific and sex-specific number of incident cases of PEM. **(B)** Age-specific and sex-specific rate of incidence of PEM per 100,000 people. **(C)** Age-specific and sex-specific number of prevalent cases of PEM. **(D)** Age-specific and sex-specific rate of incidence of PEM per 100,000 people. **(E)** Age-specific and sex-specific rate of prevalence of PEM per 100,000 people. **(F)** Age-specific and sex-specific rate of deaths due to PEM per 100,000 people. PEM, protein-energy malnutrition.

In 2021, the burden of PEM in China showed distinct age- and sex-related disparities, with bimodal peaks in both the youngest and oldest populations. The incidence of PEM was highest among middle-aged adults, particularly males aged 45–59 years, who accounted for the largest number of new cases (Figure 1A). However, children under 5 years also exhibited strikingly high age-specific incidence rates, reflecting a critical vulnerability in early childhood, especially among boys (Figure 1B). The overall prevalence followed a similar distribution, with higher burdens among middle-aged and older adults and consistently greater rates in males across all age groups (Figures 1C, D). PEM-related deaths were most concentrated at the extremes of age (the < 5 and ≥80-year groups) with mortality rates rising sharply among the older population, particularly males (Figures 1E, F). The DALYs burden mirrored these trends, peaking in children under 5 and adults aged 80–94, with males bearing a disproportionate share (Supplementary Figures 1A, B). YLDs were relatively limited but primarily occurred in middle-aged males aged 40–59 years, while females showed minimal non-fatal impact (Supplementary Figures 1C, D). YLLs dominated the DALY composition, driven by premature mortality in both the youngest and oldest populations, with a consistently greater loss in males (Supplementary Figures 1E, F).

### Temporal trends in the burden of PEM in China, 1990–2021

**Figure 2:**
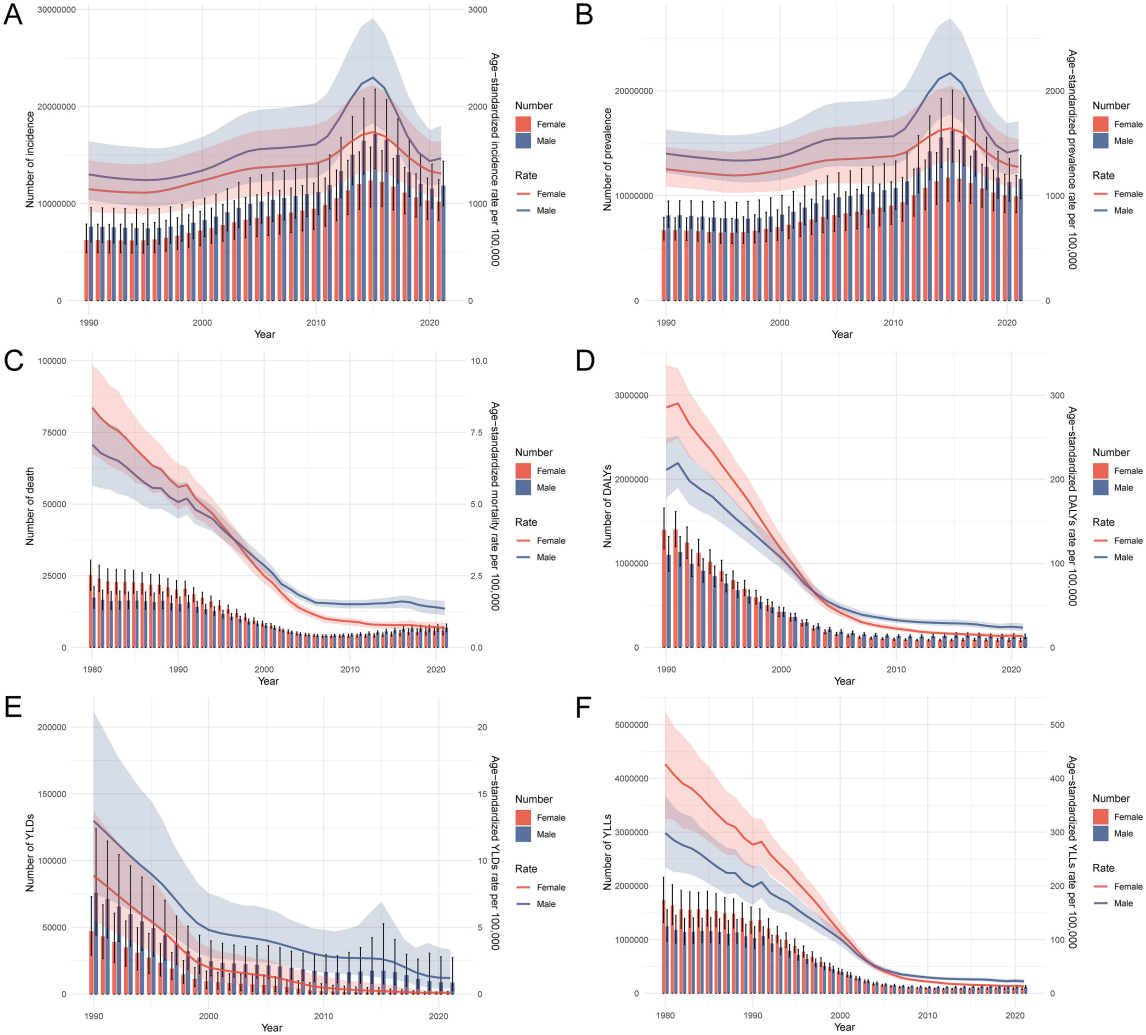
Trends in the burden of PEM in China from 1990 to 2021. **(A)** Trends in the number and age-standardized rate of incidence of PEM. **(B)** Trends in the number and age-standardized rate of prevalence of PEM. **(C)** Trends in the number and age-standardized rate of deaths due to PEM. **(D)** Trends in the number and age-standardized rate of DALYs due to PEM. **(E)** Trends in the number and age-standardized rate of YLDs due to PEM. **(F)** Trends in the number and age-standardized rate of YLLs due to PEM. PEM, protein-energy malnutrition; DALYs, disability-adjusted life years; YLDs, years lived with disability; YLLs, years of life lost.

Between 1990 and 2021, the burden of PEM in China underwent notable temporal fluctuations, with pronounced sex-specific differences. The incidence of PEM increased markedly after 2010, reaching a peak around 2015, followed by a decline in subsequent years. This pattern was more evident in males, who exhibited a sharper rise and fall, whereas changes among females were more gradual (Figure 2A). A similar trend was observed in prevalence, which peaked in 2015 and then declined, again with a steeper curve in males (Figure 2B). Mortality showed an initial decline until ~2005, after which the number of deaths gradually increased. Notably, females had higher PEM-related deaths before 2005, but this trend reversed thereafter, with males experiencing higher mortality (Figure 2C). The overall burden, as measured by DALYs, declined steadily over the study period, with males consistently bearing a higher burden from 2002 onwards (Figure 2D). YLDs decreased slightly and then stabilized, with higher rates observed in males (Figure 2E). YLLs, reflecting premature mortality, also showed a downward trend with stabilization in recent years. Initially, females had higher YLLs, but from 2004 onward, the burden shifted predominantly to males (Figure 2F). These trends reflect shifting epidemiological dynamics, with critical inflection points around 2010 and 2015, and underscore the need for sex-sensitive interventions to address the evolving burden of PEM.

### Comparative trends in the age-standardized burden of PEM in China and globally, 1990–2021

**Figure 3:**
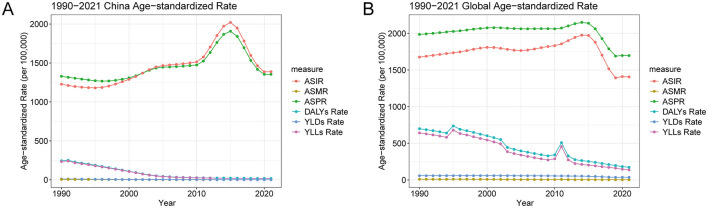
Global and China trends in the age-standardized rates of PEM from 1990 to 2021. **(A)** Global trends in the age-standardized rates of PEM. **(B)** China-specific trends in the age-standardized rates of PEM. PEM, protein-energy malnutrition.

Between 1990 and 2021, China experienced substantial declines in the age-standardized burden of PEM, particularly in mortality and premature death, with patterns largely mirroring but often exceeding global improvements. As shown in Table 2 and Figure 3, China's age-standardized mortality rate fell markedly from 5.23 to 0.91 per 100,000 population (a 4.99-point reduction), while the DALY rate dropped from 245.03 to 17.54, driven primarily by an 88% decline in YLLs. These declines were greater than those observed globally, where age-standardized mortality and DALY rates decreased by 4.06 and 4.28 points, respectively. Notably, YLDs in China also decreased by 8.95 points, reflecting a reduction in non-fatal PEM burden, whereas the global decline in YLDs was more modest. Despite these improvements, China exhibited a slight increase in age-standardized incidence (from 1,227.5 to 1,387.88 per 100,000) and a minor, non-significant rise in prevalence, contrasting with consistent global declines in both metrics. These diverging trends suggest that while PEM-related mortality and disability have been effectively reduced in China, the underlying incidence remains elevated, highlighting a potential gap in early prevention and nutritional support services that warrants continued public health attention.

**Table 2:** Change of age-standardized rates in incidence, prevalence, death, DALYs, YLDs, and YLLs for PEM between 1990 and 2021 in China and global level.

**Measure**	**China**			**Global**		
	**1990**	**2021**	**Change**	**1990**	**2021**	**Change**
Incidence	1,227.5 (978.58, 1,543.96)	1,387.88 (1,133.79, 1,713.44)	0.38 (0.21 to 0.55)^*^	1,677.02 (1,363.96, 2,070.23)	1,406.21 (1,177.88, 1,691.07)	−0.56 (−0.70 to −0.41)^*^
Prevalence	1,329.24 (1,166.59, 1,544.07)	1,354.92 (1,151.65, 1,623.8)	0.04 (−0.14 to 0.22)	1,986.52 (1,827.52, 2,186.62)	1,696.86 (1,548.51, 1,892.08)	−0.50 (−0.55 to −0.45)^*^
Deaths	5.23 (4.6, 5.85)	0.91 (0.75, 1.07)	−4.99 (−5.37 to −4.61)^*^	9.44 (8.16, 11.32)	2.61 (2.29, 2.98)	−4.06 (−6.03 to −2.04)^*^
DALYs	245.03 (209.33, 284)	17.54 (14.91, 20.26)	−8.31 (−8.94 to −7.68)^*^	699.3 (594.81, 869.67)	172.38 (142.75, 204.1)	−4.28 (−5.45 to −3.09)^*^
YLDs	11 (6.87, 16.56)	0.67 (0.26, 1.79)	−8.95 (−9.63 to −8.27)^*^	57.67 (37.97, 80.08)	35.16 (22.87, 49.92)	−1.57 (−1.62 to −1.51)^*^
YLLs	234.03 (198.82, 274.65)	16.87 (14.39, 19.46)	−7.23 (−7.60 to −6.86)^*^	641.63 (537.55, 803.44)	137.22 (110.94, 165.19)	−4.78 (−7.17 to −2.32)^*^

Values in parentheses indicate 95% UIs, estimated using Monte Carlo simulations.

PEM, protein-energy malnutrition; DALYs, disability-adjusted life-years; YLDs, years lived with disability; YLLs, years of life lost.

^*^p < 0.05 (permutation test).

### Joinpoint trends in age-standardized burden of PEM in China, 1990–2021

**Figure 4:**
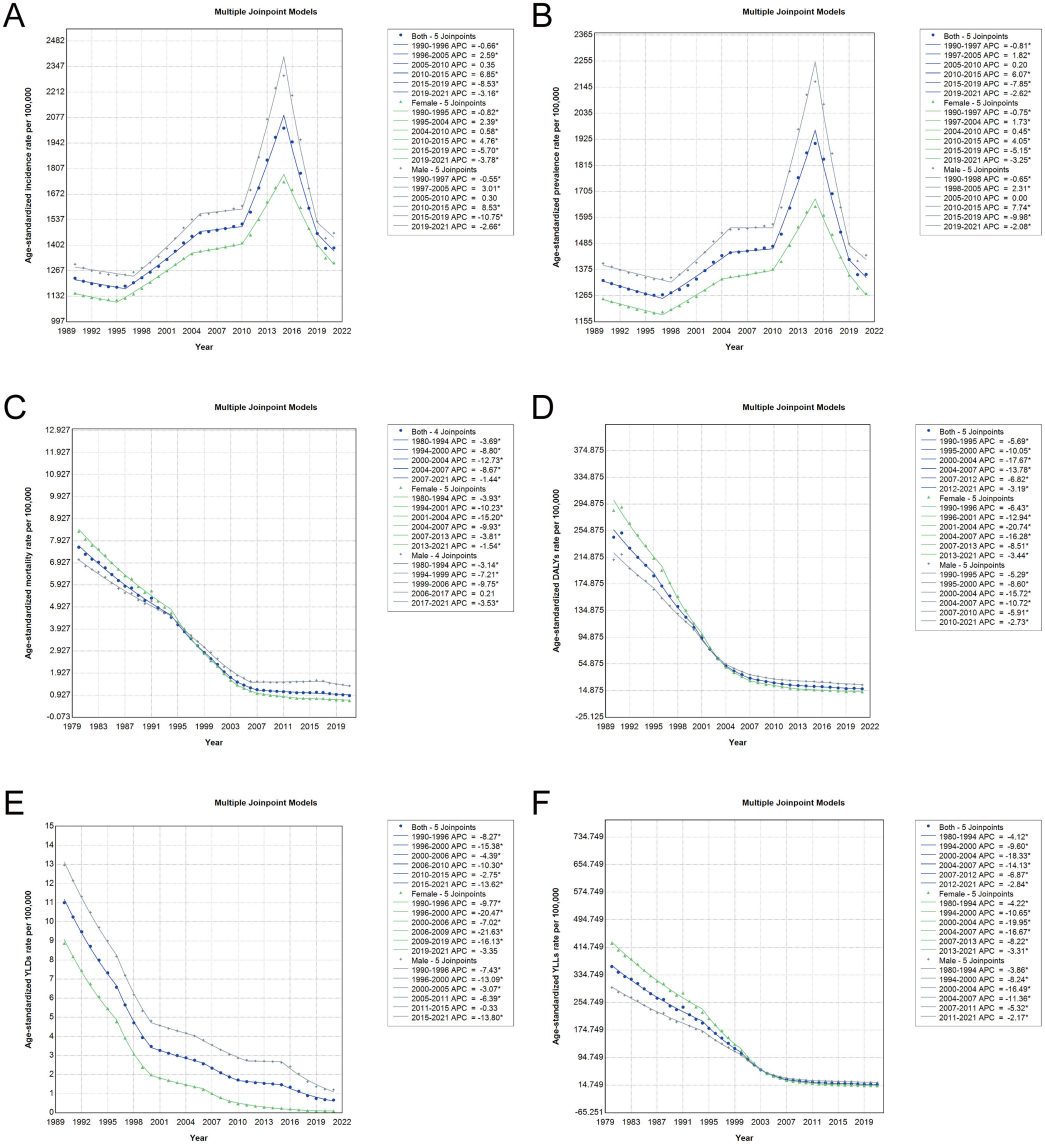
Joinpoint regression analysis of age-standardized PEM burden in China from 1990 to 2021. The analyses include data for both sexes (blue line), females (green line), and males (gray line), with asterisks representing statistically significant changes (*p* < 0.05). **(A)** Joinpoint trends in ASIR of PEM. **(B)** Joinpoint trends in ASPR of PEM. **(C)** Joinpoint trends in ASMR of PEM. **(D)** Joinpoint trends in age-standardized DALY rates of PEM. **(E)** Joinpoint trends in age-standardized YLD rates of PEM. **(F)** Joinpoint trends in age-standardized YLL rates of PEM. PEM, protein-energy malnutrition; ASIR, age-standardized incidence rate; ASPR, age-standardized prevalence rate; ASMR, age-standardized mortality rate; DALYs, disability-adjusted life years; YLDs, years lived with disability; YLLs, years of life lost.

Joinpoint regression analysis revealed distinct temporal segments in the age-standardized burden of PEM in China, marked by turning points that varied across indicators and sexes (Figure 4, Supplementary Tables 1, 2). The ASIR dropped until the mid-1990s. From 1996 to 2015, it increased, most sharply from 2010 to 2015 for both sexes (APC: 6.85% overall, 8.53% in males, 4.76% in females). After 2015, it notably declined. A similar trend was observed for the ASPR, which rose steadily until 2015 and then fell across all groups. In contrast, the ASMR declined continuously, with the steepest reductions occurring between 1994 and 2004. The age-standardized DALY rate experienced the most substantial decreases between 1995 and 2004 (APC: −17.67%), particularly in females, who exhibited more rapid improvements than males. The burden from YLDs also showed a sustained decline, especially between 1996 and 2015, although the rate of reduction slowed in recent years. YLLs mirrored DALY trends, with a sharp decline until the mid-2000s and a gradual decrease thereafter. Across nearly all metrics, males showed more pronounced fluctuations and steeper increases during rising phases, while females demonstrated more stable, consistent downward trends. These segmented patterns underscore the complex evolution of PEM burden in China, with critical periods of epidemiological transition and sex-specific dynamics shaping overall trends.

### Age, period, and cohort effects on incidence, prevalence, and DALY burden of PEM in China

**Figure 5:**
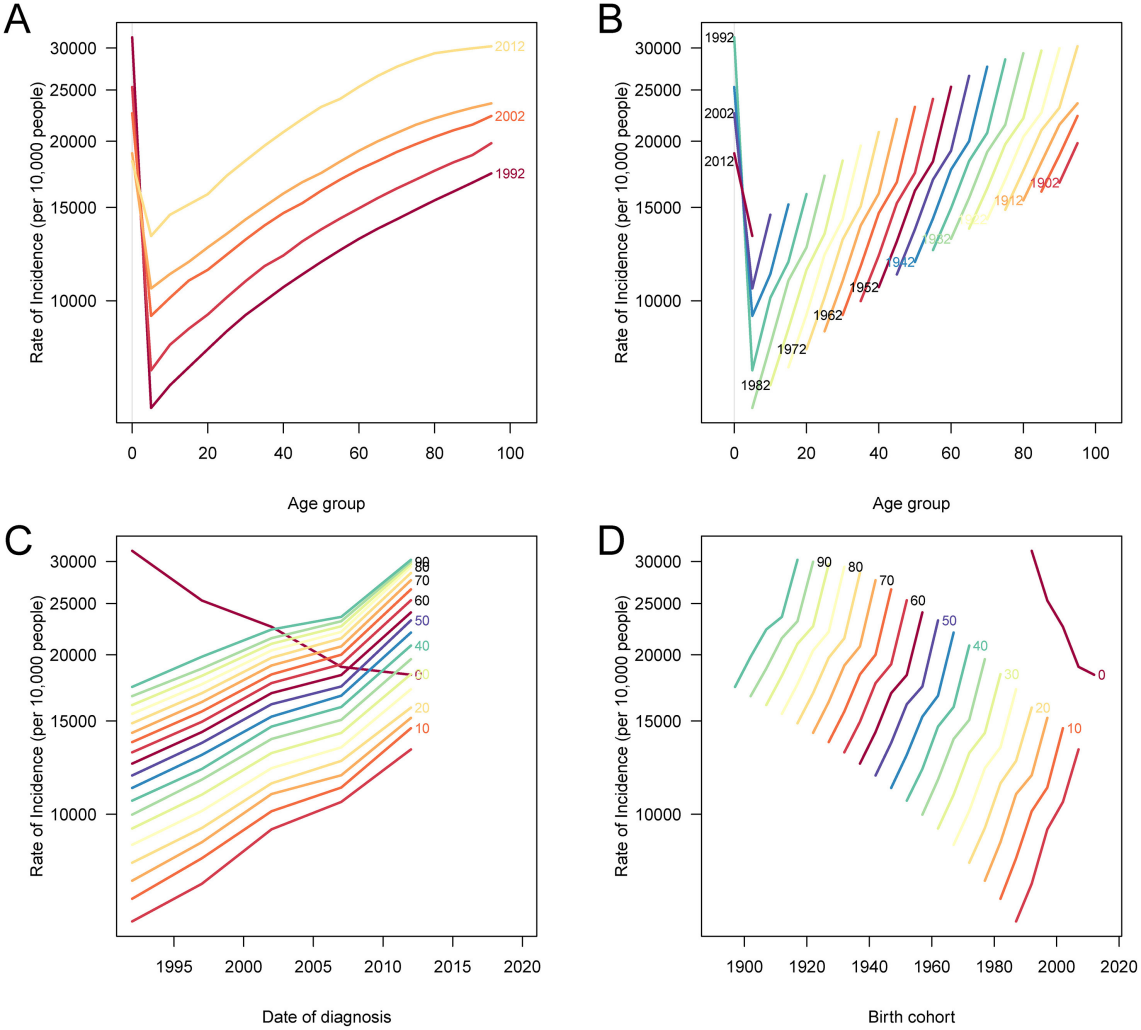
Age, period, and cohort effects on ASIR of PEM in China. **(A)** The ASIR of PEM according to time periods; each line connects the age-specific incidence for a 5-year period. **(B)** The ASIR of PEM according to birth cohorts; each line connects the age-specific incidence for a 5-year cohort. **(C)** The period-specific incidence rates of PEM according to age groups; each line connects the period-specific incidence for a 5-year age group. **(D)** The birth cohort-specific incidence rates of PEM according to age groups; each line connects the birth cohort-specific incidence for a 5-year age group. PEM, protein-energy malnutrition; ASIR, age-standardized incidence rate.

**Figure 6:**
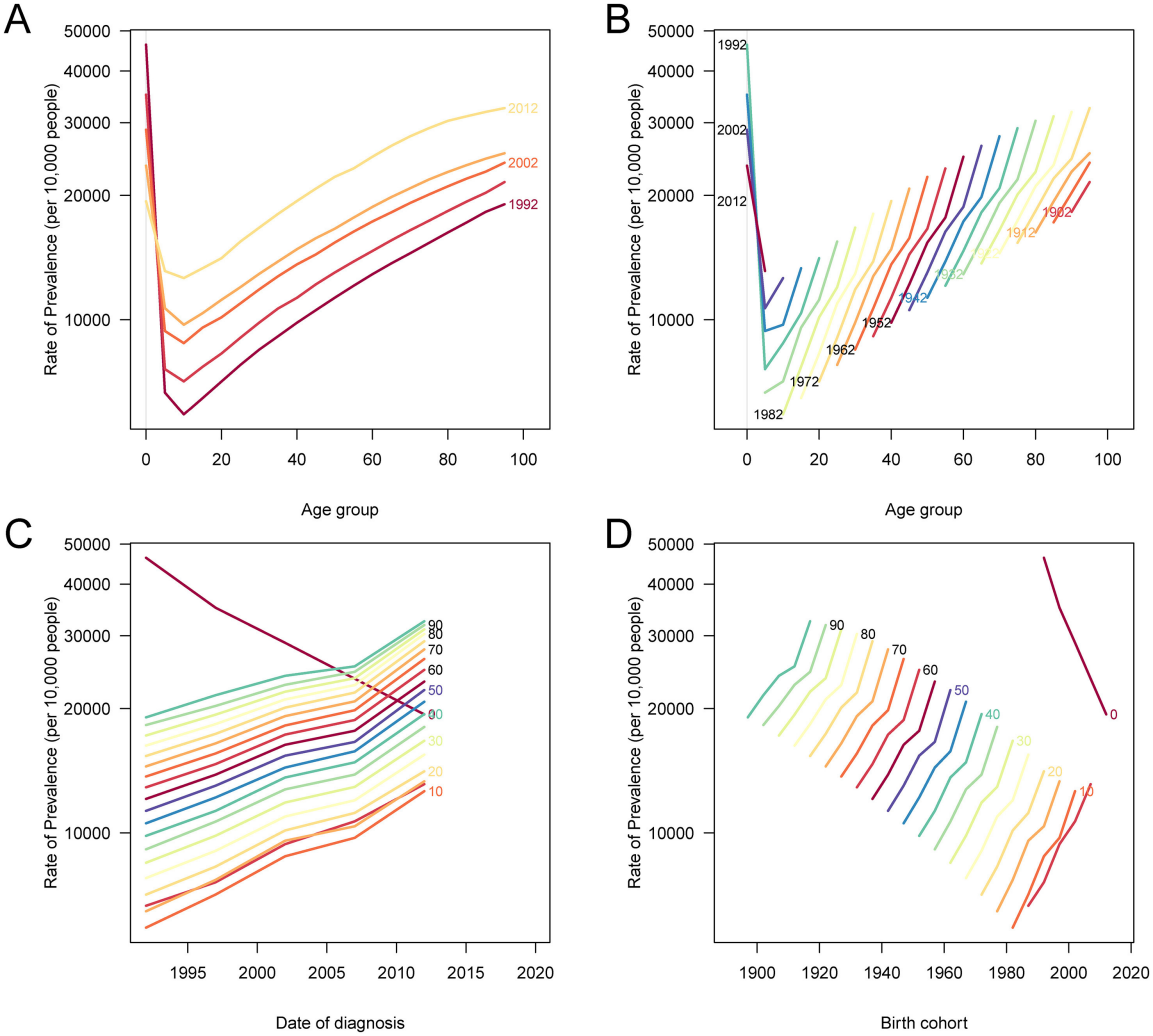
Age, period, and cohort effects on ASPR due to PEM in China. **(A)** The ASPR according to time periods; each line connects the age-specific prevalence rate for a 5-year period. **(B)** The ASPR of PEM according to birth cohorts; each line connects the age-specific prevalence rate for a 5-year cohort. **(C)** The period-specific prevalence rates of PEM according to age groups; each line connects the period-specific prevalence rate for a 5-year age group. **(D)** The birth cohort-specific prevalence rates of PEM according to age groups; each line connects the birth cohort-specific prevalence rate for a 5-year age group. PEM, protein-energy malnutrition; ASPR, age-standardized prevalence rate.

The results of the age-period-cohort analysis (Figures 5, 6, Supplementary Figure 2) revealed distinct temporal and generational patterns in the incidence, prevalence, and DALYs of PEM across the lifespan. All three indicators exhibited a bimodal distribution, with the first peak observed in the youngest age group (< 5 years), followed by a sharp decline during childhood and adolescence, and a second, more pronounced peak emerging in the older population, especially among those aged 80 years and older. For both incidence and prevalence, the burden among children under 5 has declined across recent periods and newer birth cohorts, indicating progress in early childhood nutrition (Figures 5, 6). However, among individuals older than 5 years, both indicators demonstrated increasing trends in recent periods and later birth cohorts, suggesting a growing PEM burden among older age groups. In contrast, age-standardized DALY rates consistently declined across all age groups, time periods, and birth cohorts, with the most substantial reductions occurring in the < 5-year age group (Supplementary Figure 2). These findings suggest that although the overall health impact of PEM, particularly in terms of mortality and disability, has improved over time, the rising incidence and prevalence in older populations highlight a shifting demographic risk and the need for targeted interventions across the adult life course.

### Drivers of changes in PEM incidence, prevalence and DALYs in China

**Figure 7:**
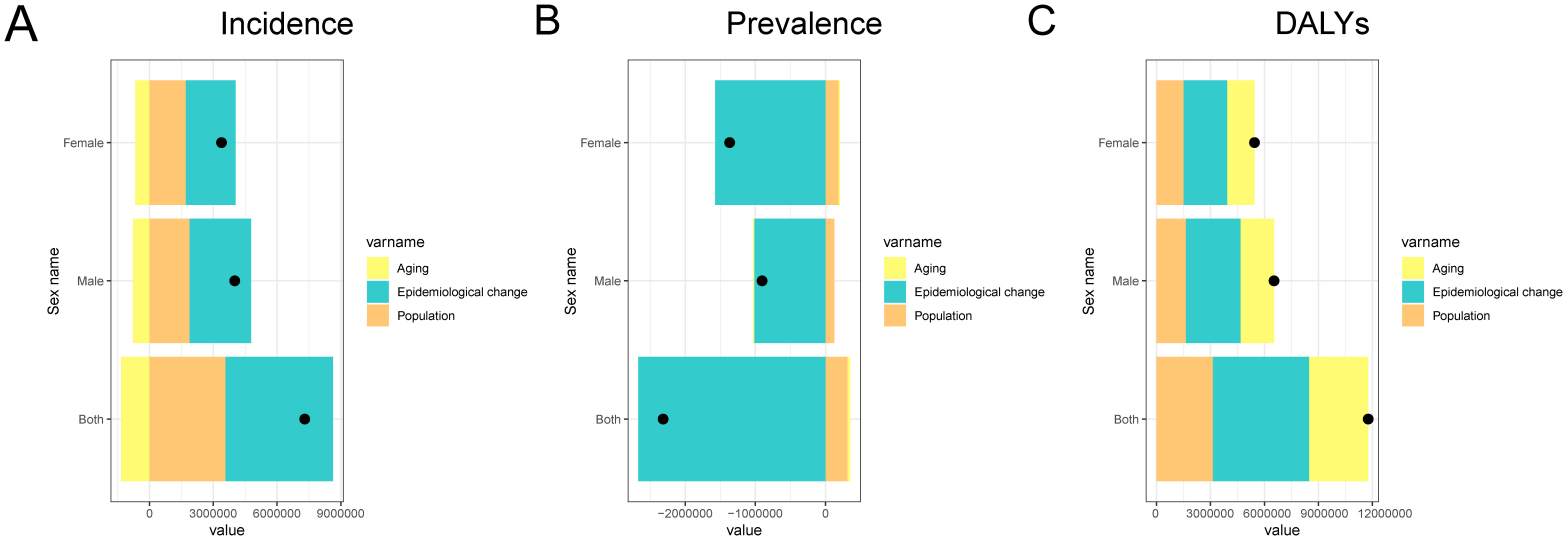
Decomposition analysis of changes in PEM burden in China from 1990 to 2021. The figure shows the contributions of aging, epidemiological changes, and population growth for both sexes (combined), males, and females. **(A)** Decomposition of factors contributing to PEM incidence; **(B)** Decomposition of factors contributing to PEM prevalence; **(C)** Decomposition of factors contributing to PEM DALYs. PEM, protein-energy malnutrition; DALYs, disability-adjusted life years.

The decomposition analysis revealed the distinct contributions of population aging, epidemiological changes, and population growth to the evolving burden of PEM in China, with clear sex-specific differences across indicators (Figure 7). For incidence (Figure 7A), population aging was associated with a reduction in both males and females, while epidemiological changes and population growth contributed to increases, leading to an overall rise in incidence. These effects were more pronounced in males. For prevalence (Figure 7B), population aging had contrasting effects: it was linked to an increase in females but a decrease in males. Epidemiological changes contributed to a reduction in prevalence in both sexes, while population growth led to increases across the board. The net effect was an overall decline in prevalence, driven primarily by the influence of epidemiological change and demographic expansion, particularly among females. In the case of DALYs (Figure 7C), all three components (aging, epidemiological changes, and population growth) contributed to an increased burden in both sexes, with a marginally higher impact observed in males. These findings suggest that while progress in health systems and nutrition may be curbing the prevalence of PEM, especially in younger populations, the ongoing effects of aging and expanding population size, along with changes in diagnostic and health-seeking behaviors, are sustaining or increasing the broader PEM burden in China.

### Projections of PEM incidence, prevalence, and DALYs in China through 2030 based on BAPC modeling

**Figure 8:**
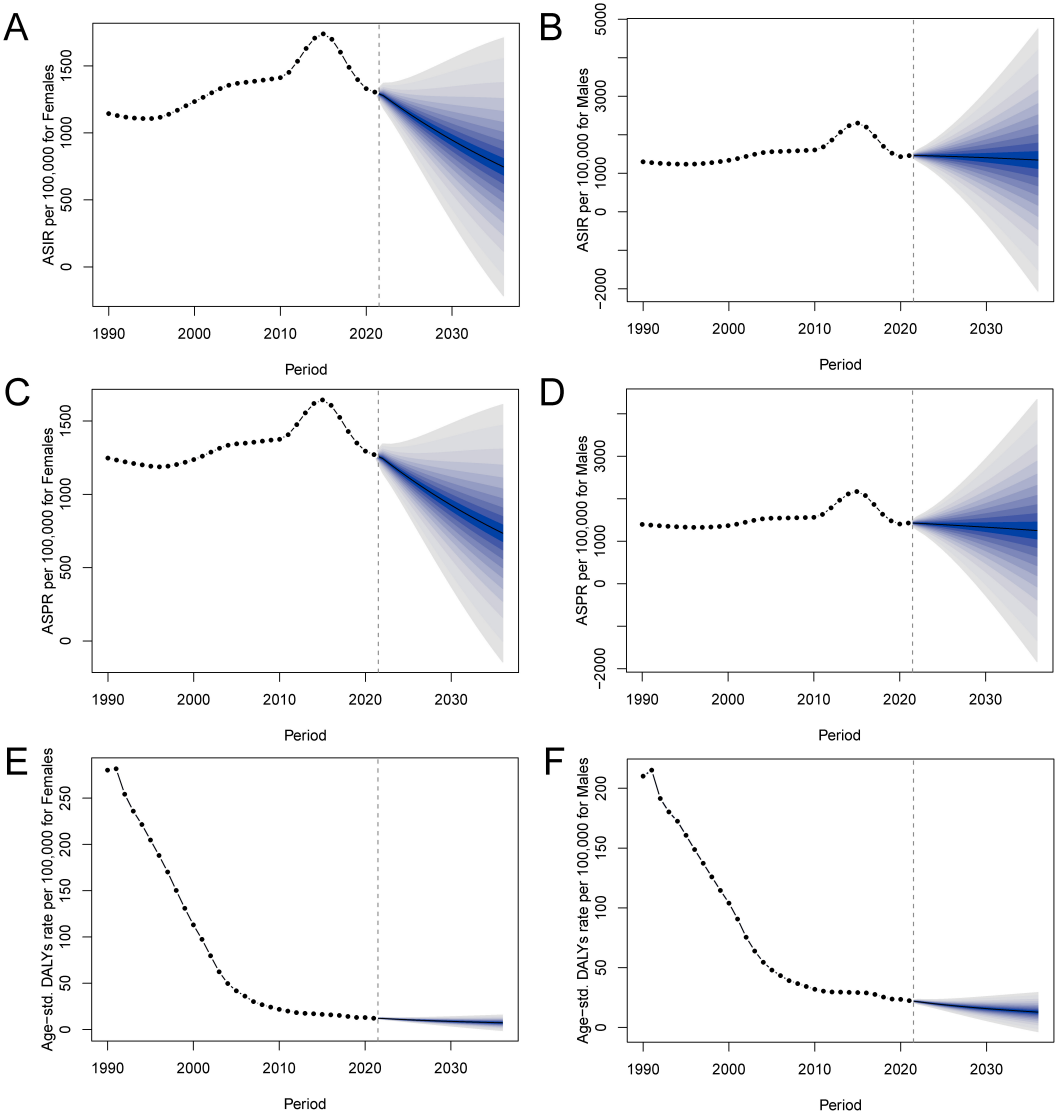
BAPC model projections of PEM incidence, prevalence, and DALY rates in China for males and females. Shaded areas around the lines represent 95% Bayesian credibility intervals. **(A)** Female incidence rates; **(B)** Male incidence rates; **(C)** Female prevalence rates; **(D)** Male prevalence rates; **(E)** Female DALY rates; **(F)** Male DALY rates. PEM, protein-energy malnutrition; BAPC, Bayesian age-period-cohort; DALYs, disability-adjusted life years.

Figure 8 presents the BAPC projections for incidence, prevalence, and DALY rates of PEM in China through 2030 for both sexes. The analysis reveals that while both males and females are expected to experience declines across all three indicators, the reductions are projected to occur more rapidly in females. For incidence, both sexes show a downward trajectory, but the decline is steeper in females, contributing to a gradual narrowing of the sex gap over time (Figures 8A, B). A similar pattern is observed in prevalence, where female rates are projected to decrease more substantially than those of males, indicating a more pronounced reduction in the female PEM burden (Figures 8C, D). Trends in DALYs mirror these findings, with females showing a faster rate of decline compared to males (Figures 8E, F). Despite overall improvements, males are projected to retain higher incidence, prevalence, and DALY rates than females throughout the projection period, although the gap between the sexes is expected to gradually diminish as the burden on females decreases more sharply.

## Discussion

This study provides a comprehensive assessment of the long-term trends and future projections of PEM in China from 1990 to 2021, using data from the GBD 2021. We found that while the age-standardized mortality and DALY rates of PEM have significantly declined over the past three decades, the all-age incidence and prevalence have remained high, particularly among males and older adults. The burden of PEM showed a distinct bimodal age distribution, with peaks in children under five and adults aged 80 years and above. Temporal trends revealed critical inflection points around 2010–2015, with a notable rise in incidence and prevalence followed by a steady decline. Comparative analysis showed that China achieved greater reductions in mortality and DALYs than global averages, though the rise in age-standardized incidence was unique to China. Joinpoint and age-period-cohort analyses highlighted increasing vulnerability in recent birth cohorts and older age groups. Decomposition analysis indicated that population aging contributed to divergent effects on PEM burden by sex, while population growth and epidemiological transitions played key roles in shaping current trends. Bayesian projections suggest that although incidence, prevalence, and DALYs are expected to continue declining through 2030, males will likely maintain a higher burden than females.

During 2010–2015, we observed a marked increase in PEM incidence and prevalence in China, which peaked in 2015 before declining thereafter. This transient rise may be linked to rapid urbanization and economic growth during that period, which substantially altered dietary behaviors across the population. Studies have shown that China experienced a dramatic shift away from traditional diets rich in fiber and complex carbohydrates toward high-fat, high-energy-density, and low-fiber dietary patterns, particularly among low-income urban residents (32). Increased consumption of edible oils, refined grains, and processed animal-source foods, combined with reduced intake of coarse grains and tubers, contributed to an emerging “double burden” of malnutrition, coexisting undernutrition, and overnutrition (33). Following 2015, the gradual decline in PEM burden likely reflects the implementation of national nutrition improvement strategies and strengthened public health interventions targeting vulnerable groups (34). Despite overall improvements, children under five years of age continue to bear a disproportionate burden, with persistently high incidence, prevalence, and YLLs. Nutritional vulnerability in this group stems from heightened physiological demands and susceptibility to growth retardation, impaired cognitive function, and increased vulnerability to infections (35–37). PEM in early childhood is closely linked to adverse long-term outcomes including stunting, impaired cognitive development, and elevated mortality risk (38). Key risk factors include insufficient breastfeeding, inappropriate complementary feeding, recurrent infections, poverty, and limited maternal education (39). While the COVID-19 pandemic did not visibly disrupt national PEM trends in 2020–2021, its potential effects, such as strained healthcare access and food insecurity, merit continued surveillance.

In individuals aged 80 years and above, a pronounced rise in mortality, DALYs, and YLLs related to PEM was observed. Older adults are particularly vulnerable to malnutrition due to multiple age-associated challenges, including diminished appetite, dental issues, swallowing difficulties, and chronic conditions that impair nutrient absorption and metabolism (40). The substantial burden of PEM in this group underscores the urgency of age-specific nutritional interventions to reduce health risks. Malnutrition in later life contributes to increased frailty, greater susceptibility to infections, prolonged hospital stays, and escalating healthcare expenditures. It also leads to a decline in functional capacity and a reduction in overall quality of life (41, 42). Evidence from a 6-year cohort study demonstrated that severe malnutrition significantly elevates all-cause mortality among those aged 60–79 years (43), reinforcing the need for targeted prevention strategies. Contributing factors to PEM in older adults include chronic illness, reduced food intake, oral health problems, limited mobility, social isolation, and restricted access to nutrient-rich foods (44). Effective intervention strategies should prioritize tailored nutritional support, chronic disease management, and the provision of integrated social and medical services that address the unique challenges faced by the older population.

The decomposition analysis offers valuable insights into the underlying drivers of trends in PEM morbidity and mortality. A decline in morbidity associated with aging may be attributed to survivorship bias, enhanced healthcare availability, and age-specific nutritional interventions that improve resilience in the older population (45). Older individuals who survive into advanced age often benefit from better health conditions and more consistent nutritional care. Advances in medical services and the implementation of nutrition-focused programs targeting older adults have likely reinforced this pattern. In contrast, mortality outcomes revealed that both population aging and growth have contributed to increased deaths from PEM, reflecting the expanding size of the older demographic. Positive shifts in healthcare infrastructure and chronic disease management, particularly among women, have helped lower mortality through improved epidemiological conditions. However, epidemiological changes also contributed to an increase in PEM incidence, likely due to rising chronic disease prevalence, longer survival of vulnerable individuals, and greater clinical recognition and diagnosis of PEM cases. Notably, while PEM prevalence has risen recently, likely due to aging, urbanization, and dietary shifts, decomposition results show a decreasing overall contribution of epidemiological change to prevalence. This apparent contradiction may be explained by improved management of PEM cases and a shift toward earlier detection and treatment, which can reduce the average disease duration and severity. The BAPC analysis forecasted that although PEM incidence may plateau, male rates are projected to remain elevated. These findings highlight the need for sustained, gender-sensitive public health strategies. National initiatives such as the “Healthy China 2030 Blueprint” support this preventive approach (46).

This study presents several limitations that warrant consideration. First, the GBD data may vary in accuracy and consistency across regions and time, which could affect the precision of estimates. Second, the reliance on secondary data sources limits the inclusion of all relevant variables and potential confounders influencing PEM trends. Third, although the analysis provides national-level insights, it does not reflect important regional disparities. Vulnerable populations, particularly those in rural, low-income, or underserved areas, face a higher risk of developing PEM due to limited access to healthcare, nutritious food, and education. The use of national averages may obscure these local inequalities, potentially underestimating the burden in high-risk groups. Moreover, socio-economic and environmental factors such as food insecurity, maternal education, and sanitation were not accounted for in this analysis. These determinants play a critical role in shaping PEM outcomes and should be integrated into future research. While the BAPC model offers useful projections, it is inherently constrained by historical trends and assumptions, which may not capture future policy changes or shifts in exposure patterns. To strengthen future investigations, high-quality, region-specific primary data are needed, along with longitudinal studies examining early-life nutrition and its long-term health impacts. Finally, addressing sex-specific differences in PEM burden and tailoring interventions to the unique needs of different demographic and geographic subgroups will be essential for equitable public health planning.

## Conclusions

This study provides a comprehensive analysis of the trends and future projections of PEM in China, underscoring the ongoing burden on vulnerable populations such as children under 5 and the older population. While significant progress has been made in reducing the overall burden of PEM, challenges remain, particularly with regard to gender disparities and the continued impact on older adults. Future research should focus on developing and implementing targeted interventions that address the specific nutritional needs of high-risk groups. Additionally, studies investigating the underlying mechanisms driving the observed trends in PEM across different age groups, as well as the role of socioeconomic factors, will be critical for informing public health strategies. Expanding access to healthcare and nutrition services, particularly in rural and underserved areas, remains a priority. Continued efforts are essential to sustain progress and further mitigate the health impact of PEM in China.

## Data Availability

Publicly available datasets were analyzed in this study. This data can be found here: https://vizhub.healthdata.org/gbd-results/. The raw data supporting the conclusions of this article will be made available by the authors, without undue reservation.

## Supplementary material

Supplementary Figure 1 Age and sex distribution of DALY, YLD, and YLL rates of PEM in China in 2021. **(A)** Age-specific and sex-specific number of DALYs due to PEM. **(B)** Age-specific and sex-specific rate of DALYs due to PEM per 100,000 people. **(C)** Age-specific and sex-specific number of YLDs due to PEM. **(D)** Age-specific and sex-specific rate of YLDs due to PEM per 100,000 people. **(E)** Age-specific and sex-specific number of YLLs due to PEM. **(F)** Age-specific and sex-specific rate of YLLs due to PEM per 100,000 people. PEM, protein-energy malnutrition; DALYs, disability-adjusted life years; YLDs, years lived with disability; YLLs, years of life lost.

Supplementary Figure 2 Age, period, and cohort effects on age-standardized DALY rates due to PEM in China. **(A)** The age-standardized DALY rates according to time periods; each line connects the age-specific DALY rates for a 5-year period. **(B)** The age-standardized DALYs rate of PEM according to birth cohorts; each line connects the age-specific DALY rates for a 5-year cohort. **(C)** The period-specific prevalence rates of PEM according to age groups; each line connects the period-specific DALY rates for a 5-year age group. **(D)** The birth cohort-specific DALYs rate of PEM according to age groups; each line connects the birth cohort-specific DALY rates for a 5-year age group. PEM, protein-energy malnutrition; DALYs, disability-adjusted life years.

Supplementary Table 1 Joinpoint regression analysis of trends in age-standardized incidence, prevalence, mortality rates (per 100,000) by sex for PEM in China, 1990–2021.

Supplementary Table 2 Joinpoint regression analysis of trends in age-standardized DALY, YLD, and YLL rates (per 100,000) by sex for PEM in China, 1990–2021.
