# Supplementary figures and images for: Temporal trends and demographic influences on protein-energy malnutrition in China: a comprehensive analysis from 1990 to 2021
Source: Front Nutr. 2025 May 16;12:1583740. doi: 10.3389/fnut.2025.1583740 (PMC12122302; doi:10.3389/fnut.2025.1583740)

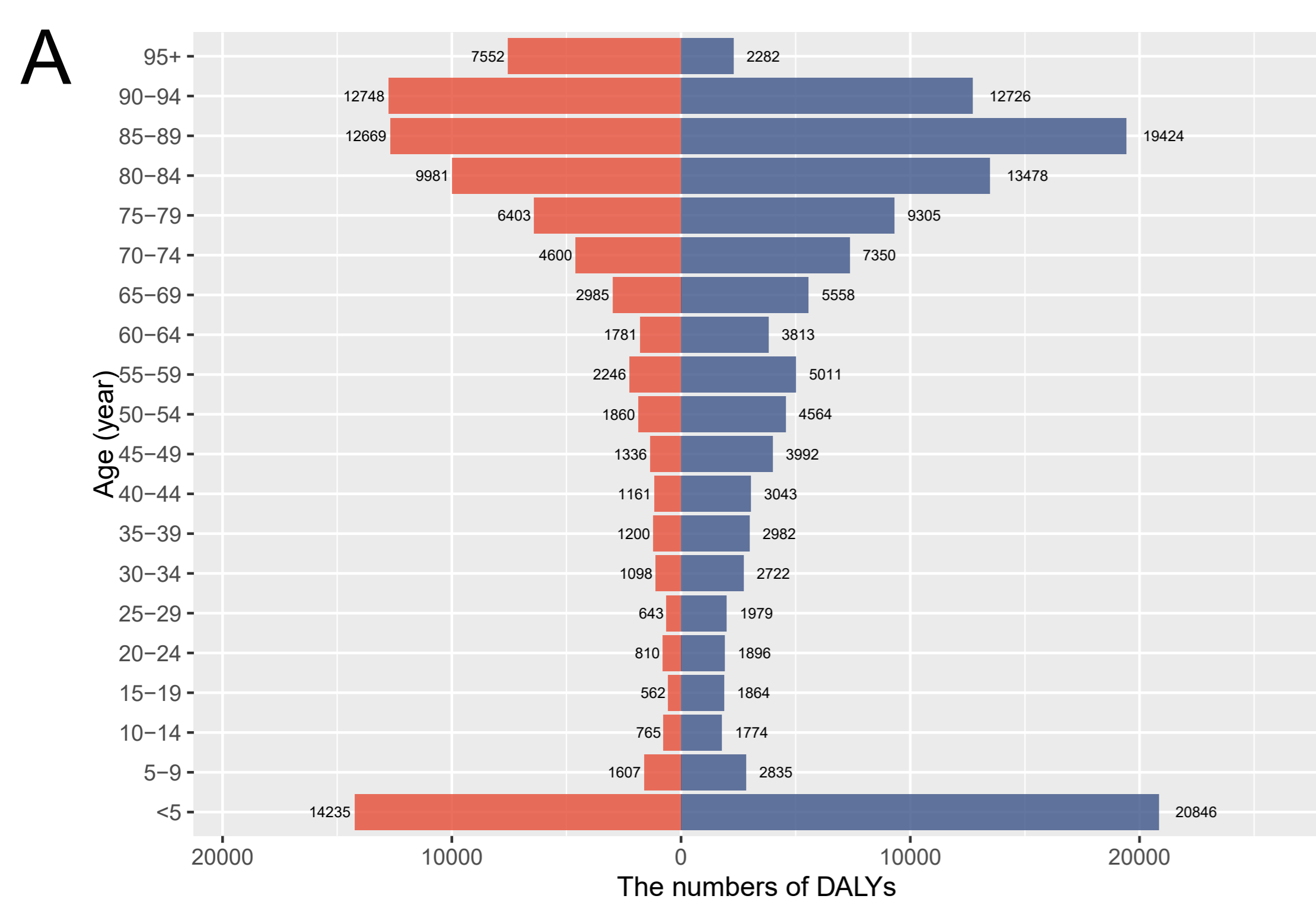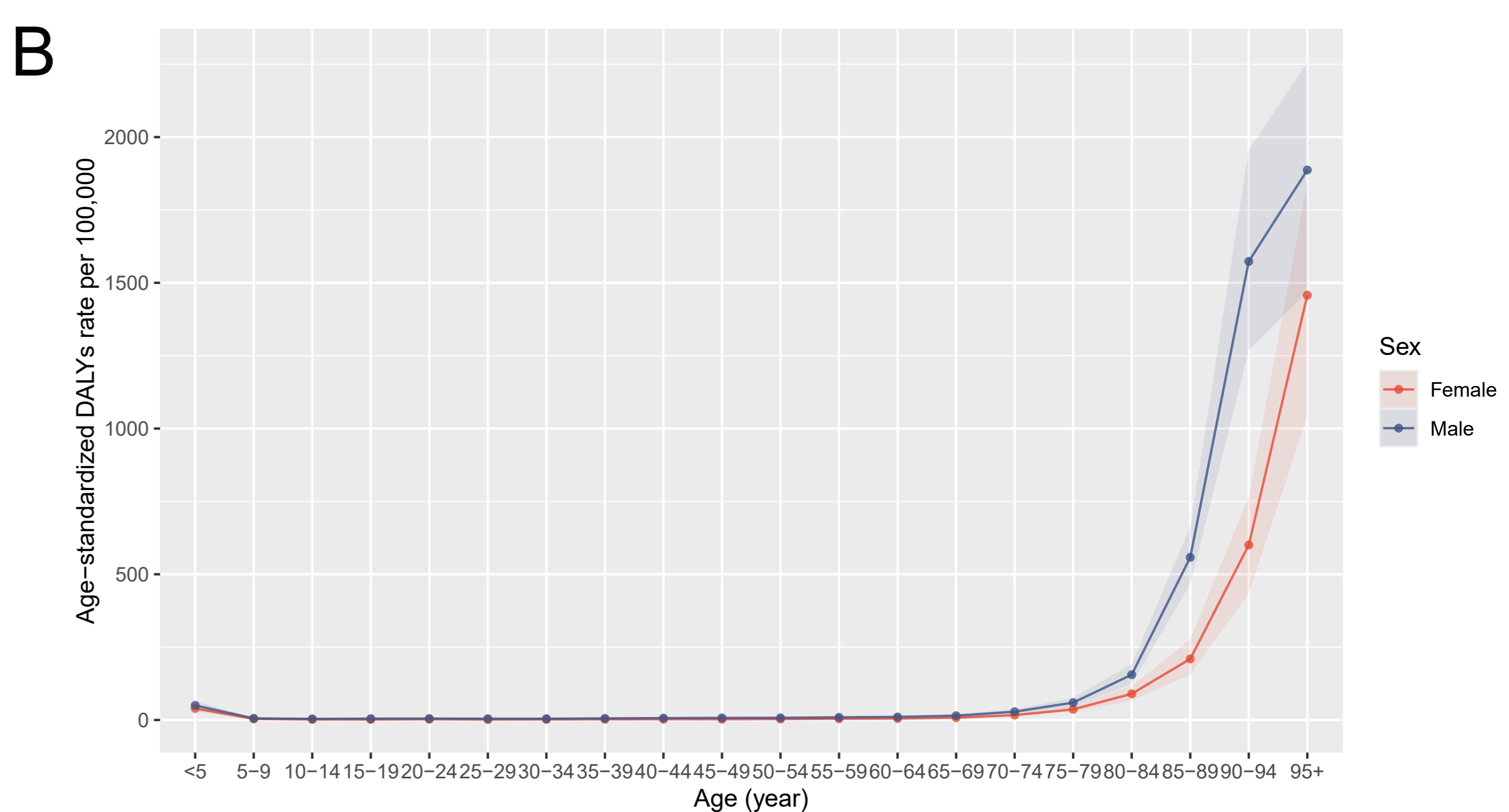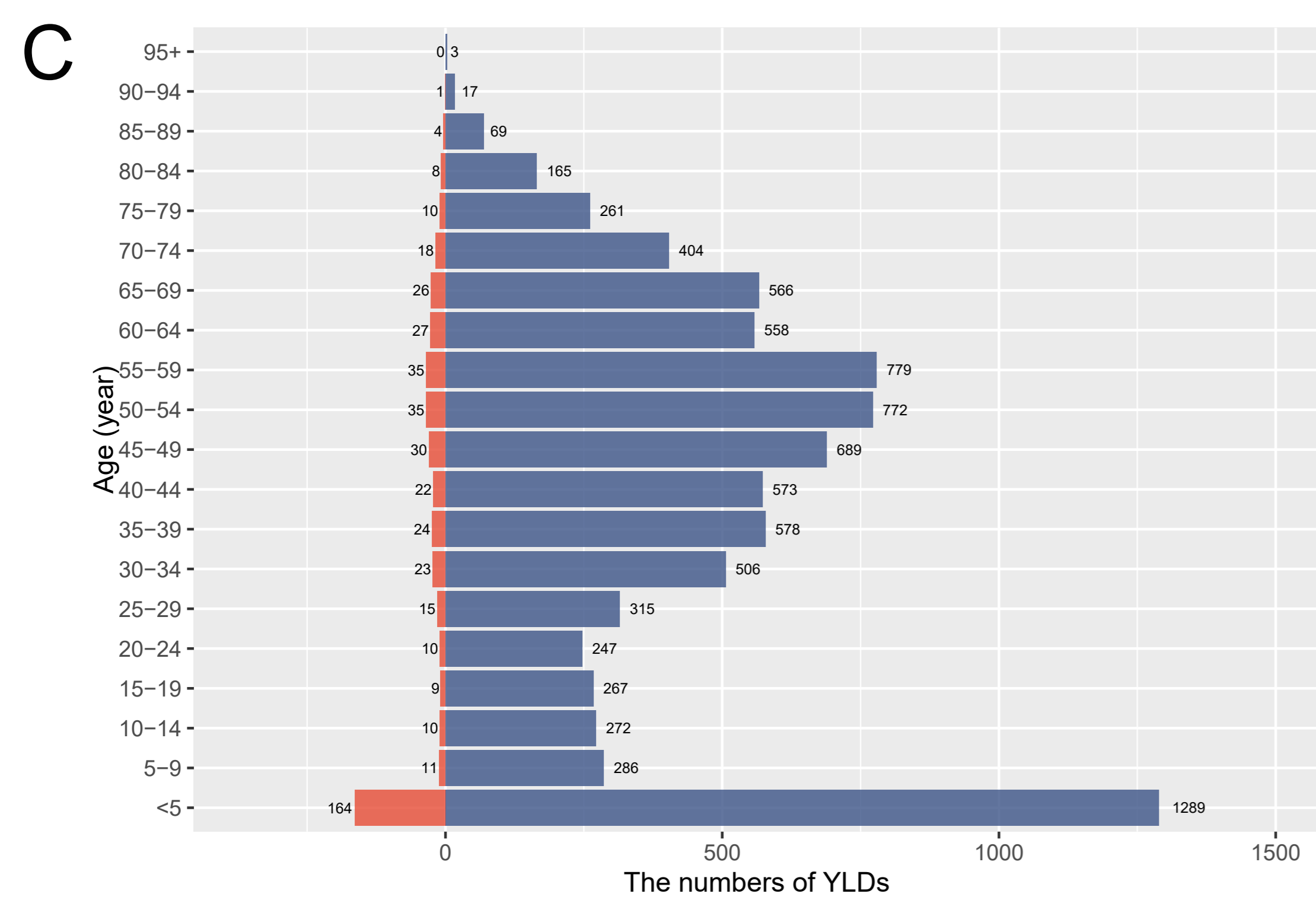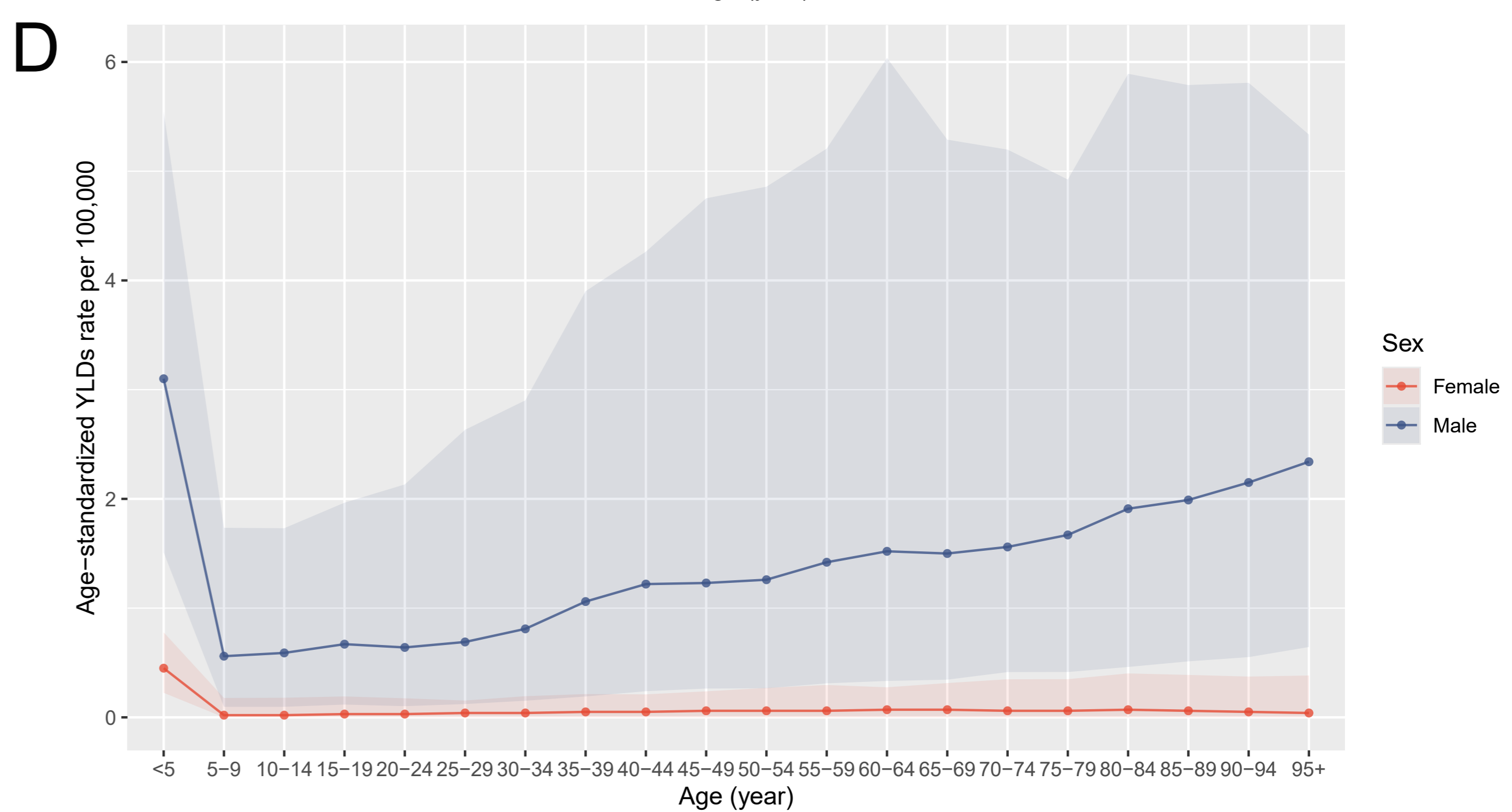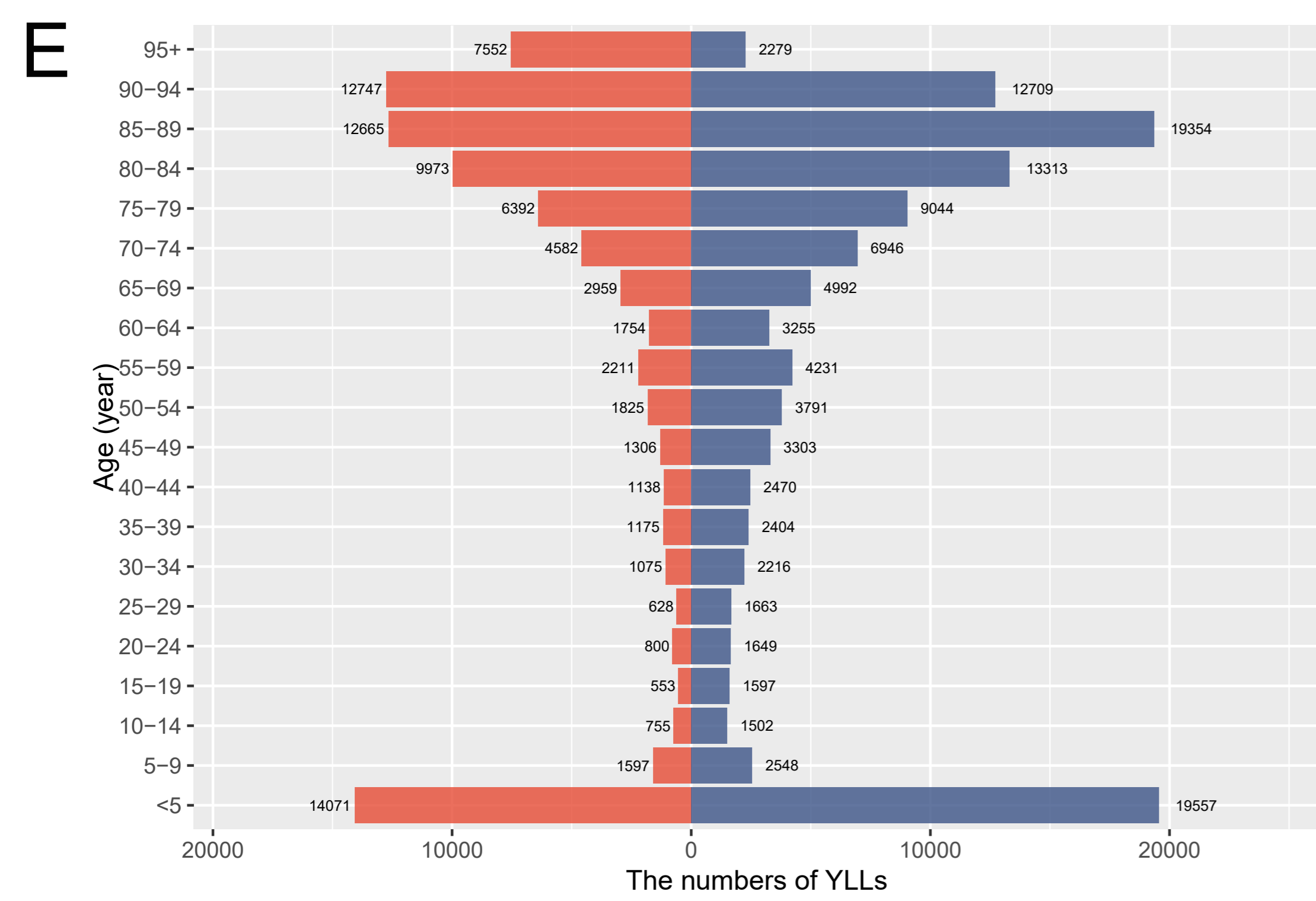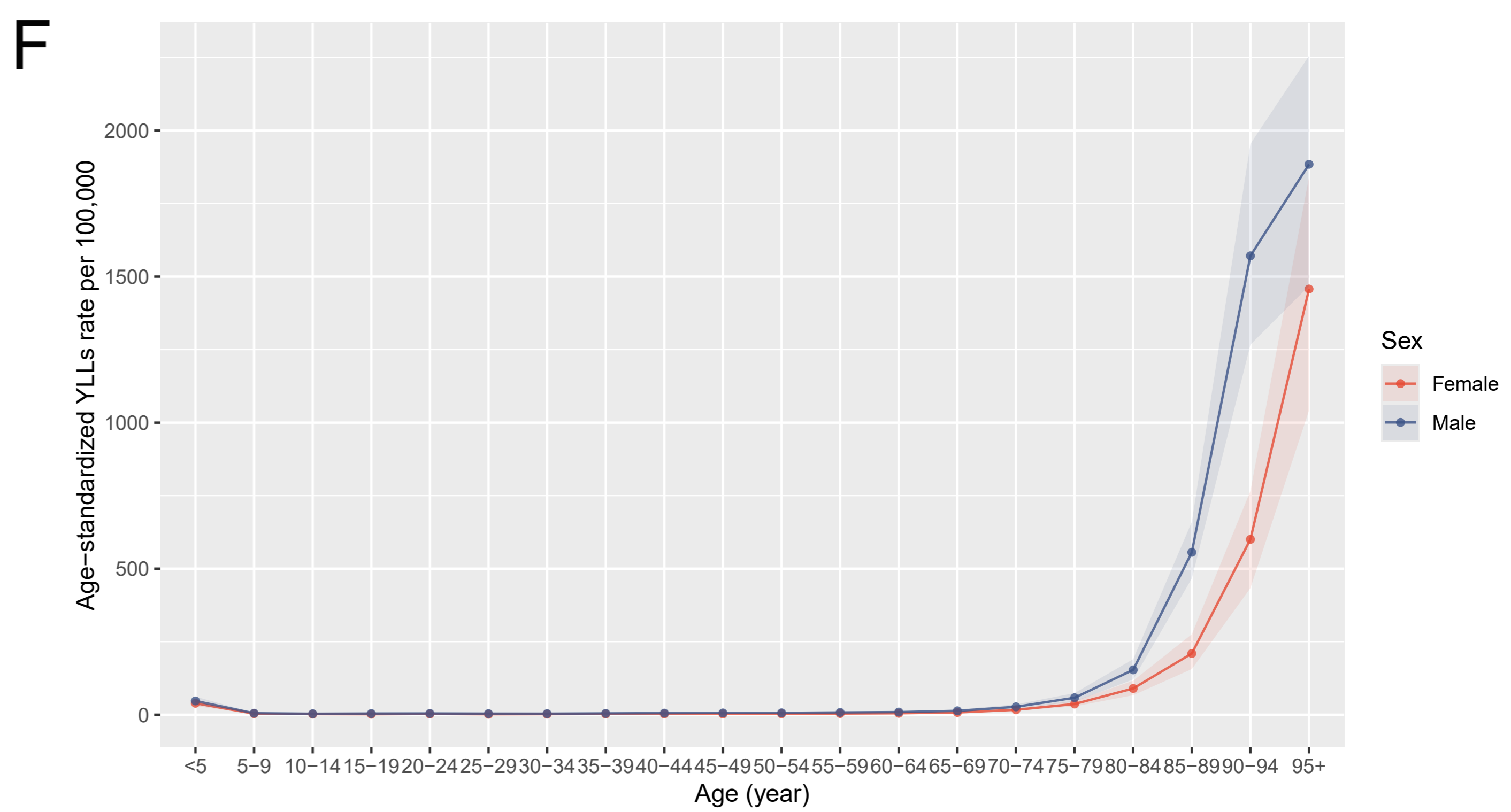

Supplement: Supplementary Figure 1 — Age and sex distribution of DALY, YLD, and YLL rates of PEM in China in 2021. (A) Age-specific and sex-specific number of DALYs due to PEM. (B) Age-specific and sex-specific rate of DALYs due to PEM per 100,000 people. (C) Age-specific and sex-specific number of YLDs due to PEM. (D) Age-specific and sex-specific rate of YLDs due to PEM per 100,000 people. (E) Age-specific and sex-specific number of YLLs due to PEM. (F) Age-specific and sex-specific rate of YLLs due to PEM per 100,000 people. PEM, protein-energy malnutrition; DALYs, disability-adjusted life years; YLDs, years lived with disability; YLLs, years of life lost. [file Data_Sheet_1.pdf]

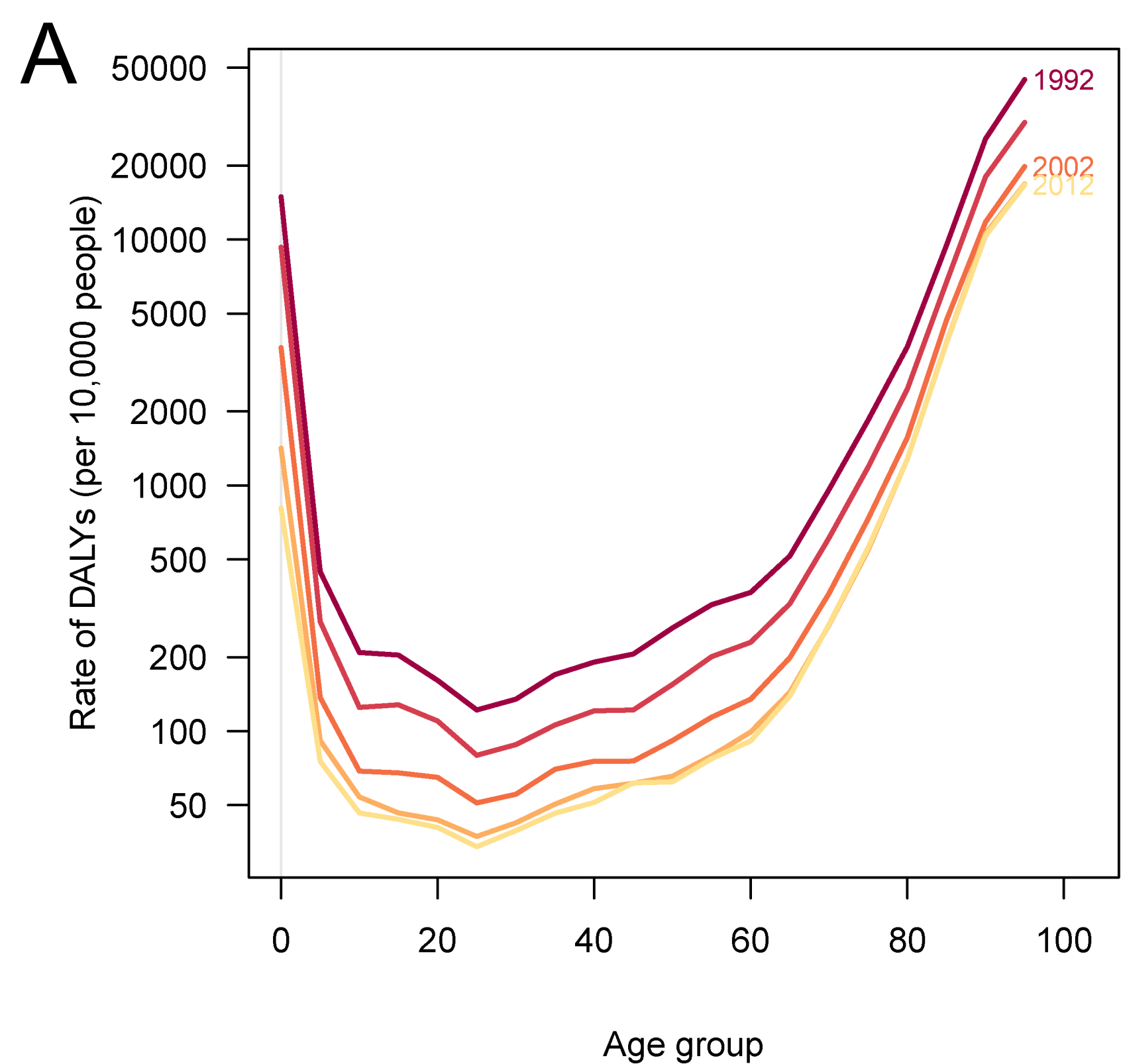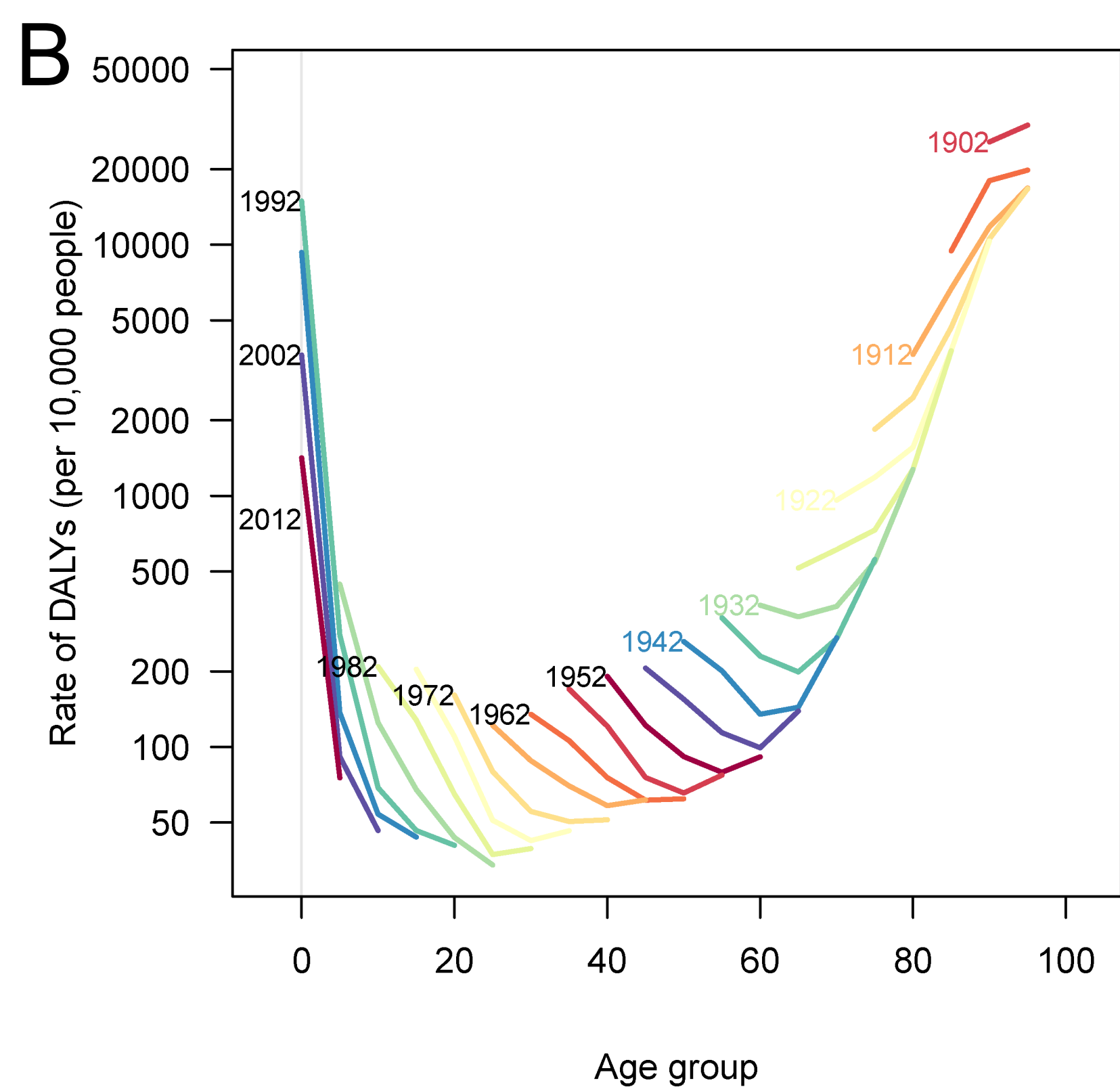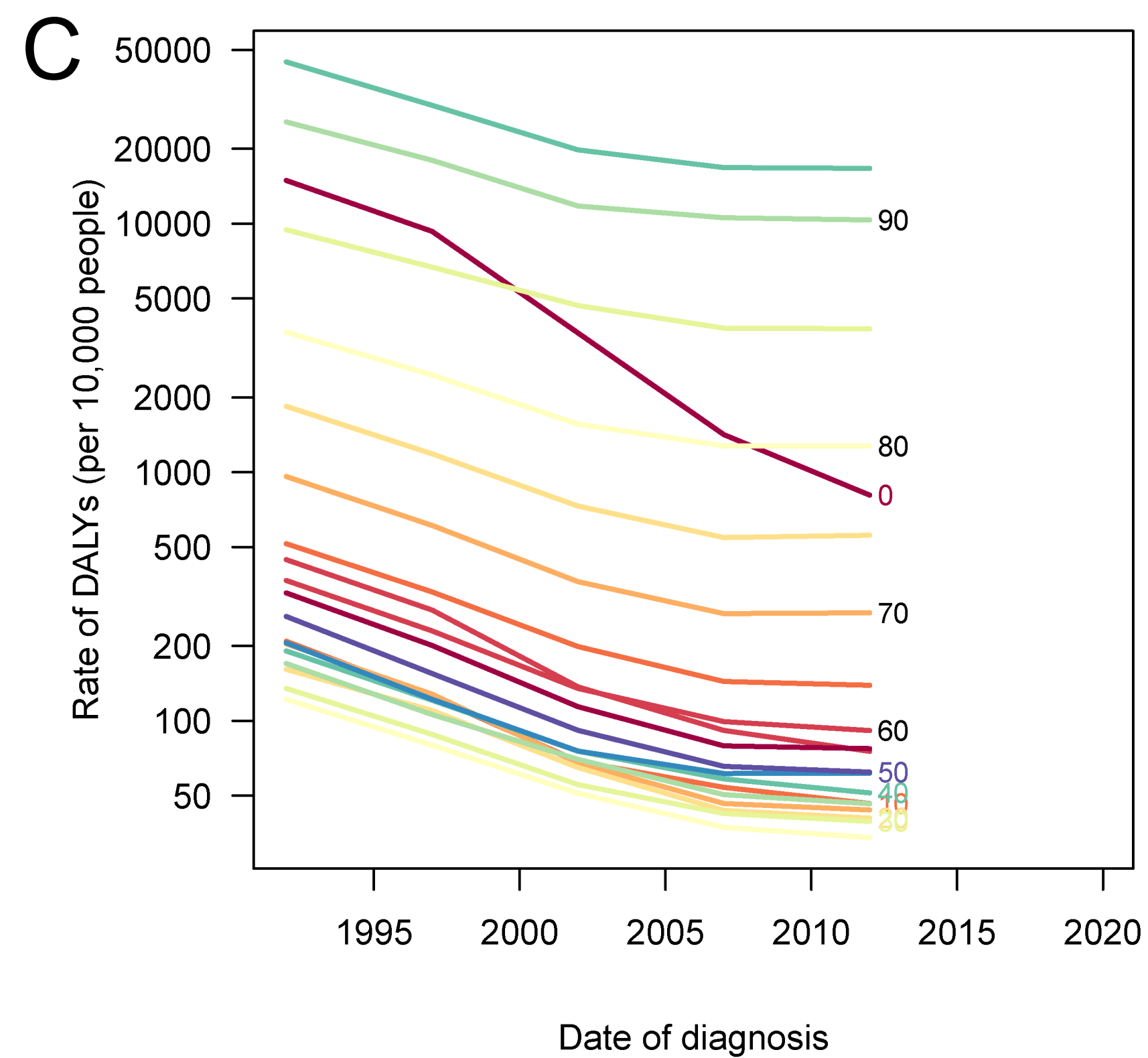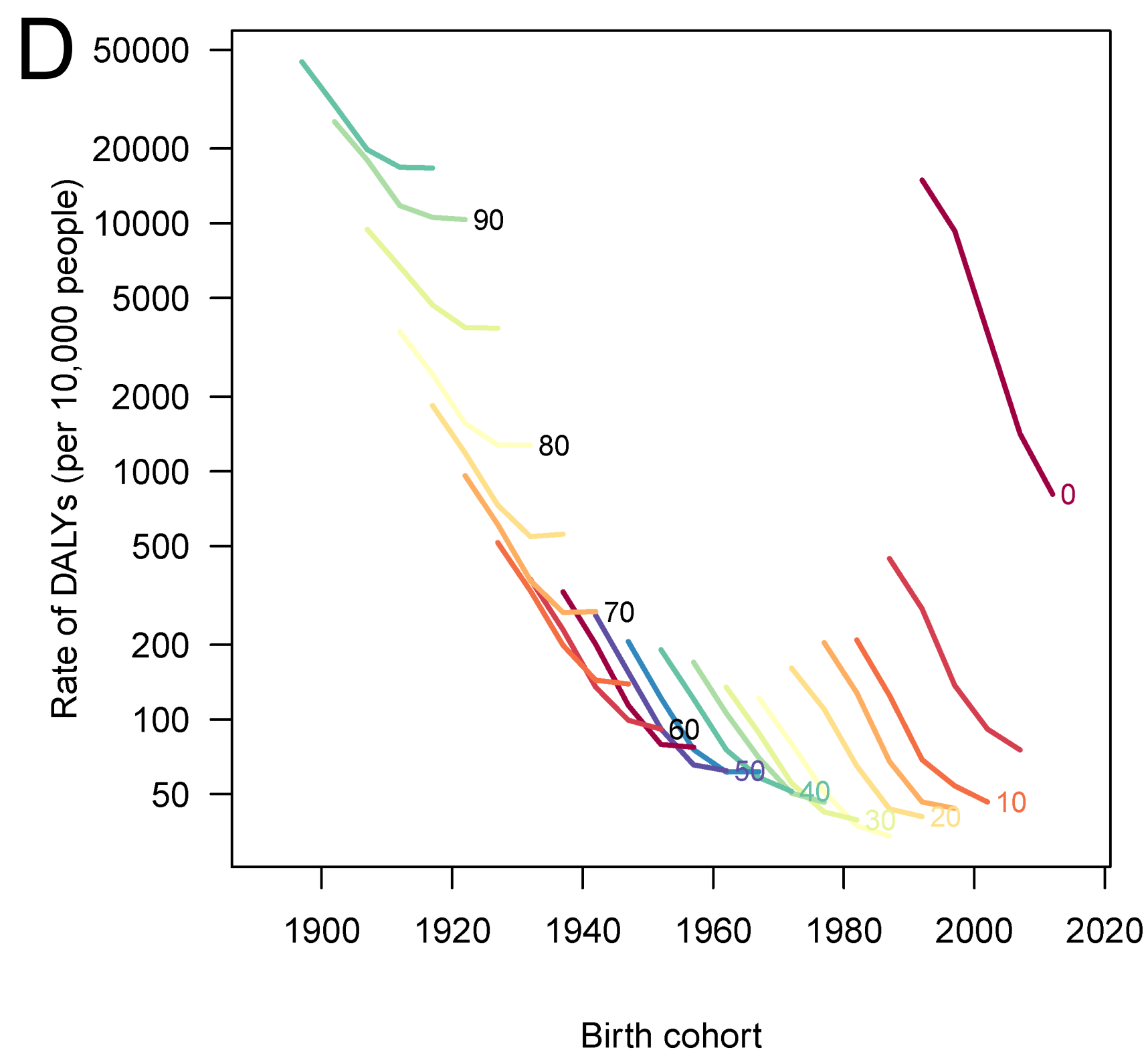

Supplement: Supplementary Figure 2 — Age, period, and cohort effects on age-standardized DALY rates due to PEM in China. (A) The age-standardized DALY rates according to time periods; each line connects the age-specific DALY rates for a 5-year period. (B) The age-standardized DALYs rate of PEM according to birth cohorts; each line connects the age-specific DALY rates for a 5-year cohort. (C) The period-specific prevalence rates of PEM according to age groups; each line connects the period-specific DALY rates for a 5-year age group. (D) The birth cohort-specific DALYs rate of PEM according to age groups; each line connects the birth cohort-specific DALY rates for a 5-year age group. PEM, protein-energy malnutrition; DALYs, disability-adjusted life years. [file Data_Sheet_2.pdf]
