# Supplementary material for: Temporal trends and demographic influences on protein-energy malnutrition in China: a comprehensive analysis from 1990 to 2021
Source: Front Nutr. 2025 May 16;12:1583740. doi: 10.3389/fnut.2025.1583740 (PMC12122302; doi:10.3389/fnut.2025.1583740)
Supplement: Supplementary Table 1 — Joinpoint regression analysis of trends in age-standardized incidence, prevalence, mortality rates (per 100,000) by sex for PEM in China, 1990–2021. [file Table_1.docx]

Supplementary Table 1. Joinpoint regression analysis of trends in age-standardized incidence, prevalence, mortality rates (per 100,000) by sex for PEM in China, 1990-2021.

|  | ASIR |  |  | ASPR |  |  | ASMR |  |  |
| --- | --- | --- | --- | --- | --- | --- | --- | --- | --- |
| Gender | Period | APC (95% CI) | AAPC (95% CI) | Period | APC (95% CI) | AAPC (95% CI) | Period | APC (95% CI) | AAPC (95% CI) |
| Both | 1990-1996 | -0.66 (-0.91 - -0.42) ^*^ | 0.38 (0.21 - 0.55) ^*^ | 1990-1997 | -0.81 (-0.97 - -0.65) ^*^ | 0.04 (-0.14 - 0.22) | 1980-1994 | -3.69 (-3.96 - -3.43) ^*^ | -4.99 (-5.37 - -4.61) ^*^ |
|  | 1996-2005 | 2.59 (2.42 - 2.76) ^*^ |  | 1997-2005 | 1.82 (1.62 - 2.02) ^*^ |  | 1994-2000 | -8.80 (-9.69 - -7.89) ^*^ |  |
|  | 2005-2010 | 0.35 (-0.14 - 0.83) |  | 2005-2010 | 0.20 (-0.31 - 0.71) |  | 2000-2004 | -12.73 (-14.58 - -10.83) ^*^ |  |
|  | 2010-2015 | 6.85 (6.33 - 7.36) ^*^ |  | 2010-2015 | 6.07 (5.50 - 6.63) ^*^ |  | 2004-2007 | -8.66 (-12.36 - -4.81) ^*^ |  |
|  | 2015-2019 | -8.53 (-9.18 - -7.86) ^*^ |  | 2015-2019 | -7.85 (-8.58 - -7.11) ^*^ |  | 2007-2021 | -1.44 (-1.70 - -1.17) ^*^ |  |
|  | 2019-2021 | -3.16 (-4.42 - -1.89) ^*^ |  | 2019-2021 | -2.62 (-3.97 - -1.26) ^*^ |  |  |  |  |
| Female | 1990-1995 | -0.82 (-1.00 - -0.64) ^*^ | 0.41 (0.31 - 0.50) ^*^ | 1990-1997 | -0.75 (-0.85 - -0.65) ^*^ | 0.05 (-0.05 - 0.15) | 1980-1994 | -3.93 (-4.17 - -3.69) ^*^ | -5.88 (-6.31 - -5.44) ^*^ |
|  | 1995-2004 | 2.39 (2.29 - 2.48) ^*^ |  | 1997-2004 | 1.73 (1.58 - 1.87) ^*^ |  | 1994-2001 | -10.23 (-10.84 - -9.62) ^*^ |  |
|  | 2004-2010 | 0.58 (0.38 - 0.77) ^*^ |  | 2004-2010 | 0.45 (0.24 - 0.67) ^*^ |  | 2001-2004 | -15.20 (-18.60 - -11.66) ^*^ |  |
|  | 2010-2015 | 4.76 (4.47 - 5.05) ^*^ |  | 2010-2015 | 4.05 (3.72 - 4.39) ^*^ |  | 2004-2007 | -9.93 (-13.40 - -6.33) ^*^ |  |
|  | 2015-2019 | -5.70 (-6.10 - -5.31) ^*^ |  | 2015-2019 | -5.15 (-5.60 - -4.70) ^*^ |  | 2007-2013 | -3.81 (-4.82 - -2.79) ^*^ |  |
|  | 2019-2021 | -3.78 (-4.50 - -3.05) ^*^ |  | 2019-2021 | -3.25 (-4.06 - -2.43) ^*^ |  | 2013-2021 | -1.54 (-2.27 - -0.81) ^*^ |  |
| Male | 1990-1997 | -0.55 (-0.84 - -0.25) ^*^ | 0.37 (0.13 - 0.61) ^*^ | 1990-1998 | -0.65 (-0.81 - -0.50) ^*^ | 0.06 (-0.14 - 0.25) | 1980-1994 | -3.14 (-3.45 - -2.83) ^*^ | -3.97 (-4.30 - -3.64) ^*^ |
|  | 1997-2005 | 3.01 (2.71 - 3.31) ^*^ |  | 1998-2005 | 2.31 (2.02 - 2.60) ^*^ |  | 1994-1999 | -7.21 (-8.52 - -5.89) ^*^ |  |
|  | 2005-2010 | 0.30 (-0.40 - 1.00) |  | 2005-2010 | 0.00 (-0.56 - 0.57) |  | 1999-2006 | -9.75 (-10.35 - -9.14) ^*^ |  |
|  | 2010-2015 | 8.53 (7.78 - 9.29) ^*^ |  | 2010-2015 | 7.74 (7.10 - 8.38) ^*^ |  | 2006-2017 | 0.21 (-0.19 - 0.61) |  |
|  | 2015-2019 | -10.75 (-11.69 - -9.79) ^*^ |  | 2015-2019 | -9.98 (-10.78 - -9.18) ^*^ |  | 2017-2021 | -3.53 (-6.00 - -1.00) ^*^ |  |
|  | 2019-2021 | -2.66 (-4.47 - -0.82) ^*^ |  | 2019-2021 | -2.08 (-3.58 - -0.55) ^*^ |  |  |  |  |

Abbreviations: PEM, Protein-energy malnutrition; AAPC, average annual percent change presented for full period; APC, annual percent change; CI, confidence interval. ^*^, *p*<0.05 (permutation test).
