# Supplementary material for: Temporal trends and demographic influences on protein-energy malnutrition in China: a comprehensive analysis from 1990 to 2021
Source: Front Nutr. 2025 May 16;12:1583740. doi: 10.3389/fnut.2025.1583740 (PMC12122302; doi:10.3389/fnut.2025.1583740)
Supplement: Supplementary Table 2 — Joinpoint regression analysis of trends in age-standardized DALY, YLD, and YLL rates (per 100,000) by sex for PEM in China, 1990–2021. [file Table_2.docx]

Supplementary Table 2. Joinpoint regression analysis of trends in age-standardized DALY, YLD, and YLL rates (per 100,000) by sex for PEM in China, 1990-2021.

|  | DALYs |  |  | YLDs |  |  | YLLs |  |  |
| --- | --- | --- | --- | --- | --- | --- | --- | --- | --- |
| Gender | Period | APC (95% CI) | AAPC (95% CI) | Period | APC (95% CI) | AAPC (95% CI) | Period | APC (95% CI) | AAPC (95% CI) |
| Both | 1990-1995 | -5.69 (-7.12 - -4.23) ^*^ | -8.31 (-8.94 - -7.68) ^*^ | 1990-1996 | -8.27 (-9.07 - -7.46) ^*^ | -8.95 (-9.63 - -8.27) ^*^ | 1980-1994 | -4.12 (-4.41 - -3.84) ^*^ | -7.23 (-7.60 - -6.86) ^*^ |
|  | 1995-2000 | -10.05 (-11.64 - -8.44) ^*^ |  | 1996-2000 | -15.38 (-17.60 - -13.10) ^*^ |  | 1994-2000 | -9.60 (-10.53 - -8.67) ^*^ |  |
|  | 2000-2004 | -17.67 (-19.78 - -15.50) ^*^ |  | 2000-2006 | -4.39 (-5.56 - -3.20) ^*^ |  | 2000-2004 | -18.33 (-20.05 - -16.57) ^*^ |  |
|  | 2004-2007 | -13.78 (-17.91 - -9.45) ^*^ |  | 2006-2010 | -10.30 (-12.93 - -7.59) ^*^ |  | 2004-2007 | -14.13 (-17.27 - -10.87) ^*^ |  |
|  | 2007-2012 | -6.82 (-8.18 - -5.44) ^*^ |  | 2010-2015 | -2.75 (-5.15 - -0.29) ^*^ |  | 2007-2012 | -6.87 (-7.92 - -5.82) ^*^ |  |
|  | 2012-2021 | -3.19 (-3.72 - -2.65) ^*^ |  | 2015-2021 | -13.62 (-15.32 - -11.88) ^*^ |  | 2012-2021 | -2.84 (-3.24 - -2.44) ^*^ |  |
| Female | 1990-1996 | -6.43 (-7.40 - -5.45) ^*^ | -9.62 (-10.32 - -8.92) ^*^ | 1990-1996 | -9.77 (-10.53 - -9.00) ^*^ | -13.59 (-14.67 - -12.50) ^*^ | 1980-1994 | -4.22 (-4.50 - -3.94) ^*^ | -8.18 (-8.57 - -7.79) ^*^ |
|  | 1996-2001 | -12.94 (-14.50 - -11.35) ^*^ |  | 1996-2000 | -20.47 (-22.63 - -18.26) ^*^ |  | 1994-2000 | -10.65 (-11.62 - -9.67) ^*^ |  |
|  | 2001-2004 | -20.74 (-24.84 - -16.41) ^*^ |  | 2000-2006 | -7.02 (-8.52 - -5.49) ^*^ |  | 2000-2004 | -19.95 (-21.72 - -18.14) ^*^ |  |
|  | 2004-2007 | -16.28 (-20.43 - -11.92) ^*^ |  | 2006-2009 | -21.63 (-28.28 - -14.36) ^*^ |  | 2004-2007 | -16.67 (-19.96 - -13.24) ^*^ |  |
|  | 2007-2013 | -8.51 (-9.50 - -7.50) ^*^ |  | 2009-2019 | -16.13 (-17.19 - -15.06) ^*^ |  | 2007-2013 | -8.22 (-9.02 - -7.41) ^*^ |  |
|  | 2013-2021 | -3.44 (-4.11 - -2.76) ^*^ |  | 2019-2021 | -3.35 (-15.09 - 10.00) |  | 2013-2021 | -3.31 (-3.86 - -2.76) ^*^ |  |
| Male | 1990-1995 | -5.29 (-6.91 - -3.65) ^*^ | -6.95 (-7.74 - -6.15) ^*^ | 1990-1996 | -7.43 (-8.28 - -6.57) ^*^ | -7.69 (-8.39 - -6.99) ^*^ | 1980-1994 | -3.86 (-4.18 - -3.54) ^*^ | -6.11 (-6.52 - -5.71) ^*^ |
|  | 1995-2000 | -8.60 (-10.42 - -6.74) ^*^ |  | 1996-2000 | -13.09 (-15.31 - -10.81) ^*^ |  | 1994-2000 | -8.24 (-9.25 - -7.22) ^*^ |  |
|  | 2000-2004 | -15.72 (-18.13 - -13.25) ^*^ |  | 2000-2005 | -3.07 (-4.71 - -1.41) ^*^ |  | 2000-2004 | -16.49 (-18.34 - -14.58) ^*^ |  |
|  | 2004-2007 | -10.72 (-15.28 - -5.92) ^*^ |  | 2005-2011 | -6.39 (-7.57 - -5.20) ^*^ |  | 2004-2007 | -11.36 (-14.66 - -7.93) ^*^ |  |
|  | 2007-2010 | -5.91 (-10.62 - -0.95) ^*^ |  | 2011-2015 | -0.33 (-4.22 - 3.73) |  | 2007-2011 | -5.32 (-7.05 - -3.55) ^*^ |  |
|  | 2010-2021 | -2.73 (-3.19 - -2.26) ^*^ |  | 2015-2021 | -13.80 (-15.42 - -12.16) ^*^ |  | 2011-2021 | -2.17 (-2.55 - -1.77) ^*^ |  |

Abbreviations: DALYs, disability-adjusted life years; YLDs, years lived with disability; YLLs, years of life lost; PEM, protein-energy malnutrition; AAPC, average annual percent change presented for full period; APC, annual percent change; CI, confidence interval. ^*^, *p*<0.05.
